# Supplementary material for: Graphene/silicon heterojunction for reconfigurable phase-relevant activation function in coherent optical neural networks
Source: Nat Commun. 2023 Oct 31;14:6939. doi: 10.1038/s41467-023-42116-6 (PMC10618201; doi:10.1038/s41467-023-42116-6)
Supplement: Supplementary file 1 — Supplementary Information [file 41467_2023_42116_MOESM1_ESM.pdf]

Supplementary Information
Graphene/silicon heterojuction for reconfigurable phase-relevant activation function in coherent optical neural networks

Chuyu Zhong[1†], Kun Liao[2†], Tianxiang Dai[2], Maoliang Wei[1], Hui Ma[1], Jianghong Wu[3,4], Zhibin Zhang[2], Yuting Ye[3,4], Ye Luo[3,4], Zequn Chen[3,4], Jialing Jian[3,4], Chulei Sun[3,4], Bo Tang[5], Peng Zhang[5], Ruonan Liu[5], Junying Li[1], Jianyi Yang[1], Lan Li[3,4], Kaihui Liu[2], Xiaoyong Hu[2,*], and Hongtao Lin[1,6,*]

[1]State Key Laboratory of Modern Optical Instrumentation, College of Information Science and Electronic Engineering, Zhejiang University, Hangzhou 310027, China.
[2]State Key Laboratory for Mesoscopic Physics, Frontiers Science Center for Nano-optoelectronics, School of Physics, Peking University, Beijing 100871, China
[3]Key Laboratory of 3D Micro/Nano Fabrication and Characterization of Zhejiang Province, School of Engineering, Westlake University, Hangzhou, Zhejiang 310024, China
[4]Institute of Advanced Technology, Westlake Institute for Advanced Study, Hangzhou, Zhejiang 310024, China
[5]Institute of Microelectronics of the Chinese Academy of Sciences, Beijing 100029, China
[6]MOE Frontier Science Center for Brain Science & Brain-Machine Integration, Zhejiang University, Hangzhou 310027, China.

† These authors contributed equally to this work.
*hometown@zju.edu, xiaoyonghu@pku.edu.cn

This Supplementary Information comprises the following Sections:

Section I - Material properties of the graphene
Section II - Fabrication process flow and morphology of our devices
Section III - Measurement setups
Section IV - Performance comparison of on-chip graphene-based/silicon-based modulators and detectors
Section V - Performance comparison of devices for optical activation function
Section VI - Statistical performance characterization of our devices
Section VII - Phase shift deduction of the ring resonator
Section VIII - Details of the optical neural network
Section IX - Common activation functions and our optical activation functions used in training
Section X - Details of the optical activation functions
Section XI - Results comparison of the networks using different activation functions
Section XII - Training results using additional optical activation functions without/with phase shift
Section XIII - Comparison of the activation maps using different activation functions and optical functions

Section I - Material properties of the graphene

The material properties of the graphene samples used in our devices were characterized and demonstrated in this section. We had performed Hall effect measurement (Ecopia HMS-5000) and Raman spectroscopy (Witec alpha300R). Graphene transferred to the 300-nm-thick silicon dioxide substrates and graphene on device with photoresist cladding were characterized.

We used the van der Pauw method to perform Hall effect measurement under magnetic field intensity of 0.535 T. The electric properties of the graphene on silicon dioxide substrate are listed in Table S1. Low resistivity and high mobility were obtained. The sign of the sheet concentration indicates a p-type doping of our sample.

Table S1. Electric properties of the graphene.

Sheet Con. (cm^{-3})	Sheet Resistance ($\Omega_{\square}$)	Resistivity ($\Omega \cdot cm$)	Conductivity ($S \cdot cm$)	Mobility (cm^2/Vs)
1.05×10^{12}	2.01×10^{3}	6.83×10^{-5}	1.46×10^{4}	2.69×10^{3}

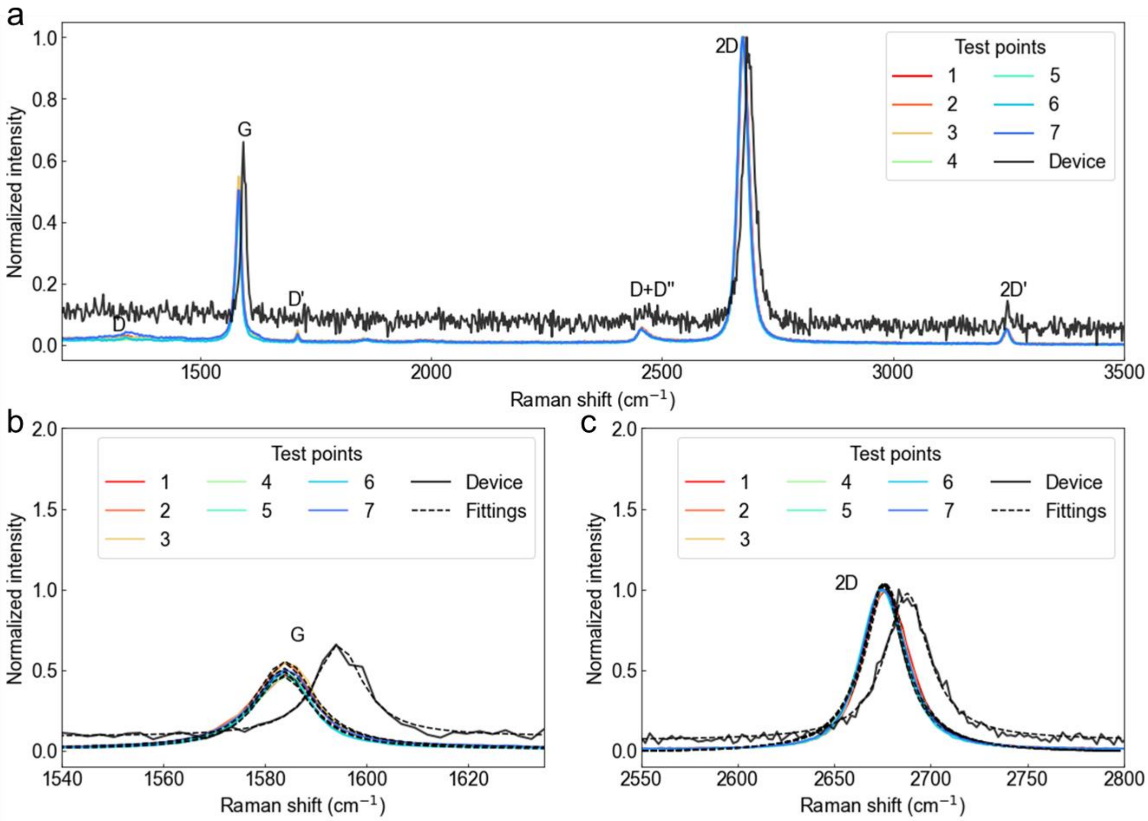

Fig. S1. Raman spectra of graphene samples on the silicon oxide substrate (test points 1 - 7) and the device. (a) The whole spectra of different samples, where different peaks are indicated. Details of the (b) G peaks and (c) 2D peaks of different samples (solid lines) and their fitted Lorentzians (dashed line)

We also performed Raman spectroscopy, which is a versatile tool for characterization of the graphene properties [S1] for more material characterization. We totally measured seven sample

points (test points 1 - 7) in silicon dioxide substrate and one sample point in the device with photoresist cladding as depicted in Fig. S1(a). Spectra (G peak and 2D peak) were fitted by Lorentz function as shown in Fig. S1(b), (c).

Firstly, it can be observed from Fig. S1 that the Raman spectra of all the samples are almost identical except that the one of the device shows peak shift and noises because of different doping, stress and extra photoresist cladding. Therefore, our graphene samples have high uniformity of quality. Secondly, the intensities of the D peaks are very low indicating that our graphene samples have few defects. In addition, as discussed in reference [S1, 2], the shape of the 2D peak is the most effective way to identify a single layer, the 2D peaks of all curves can be fitted with a single Lorentzian function (Fig. S1(c)), indicating that our sample has only one layer.

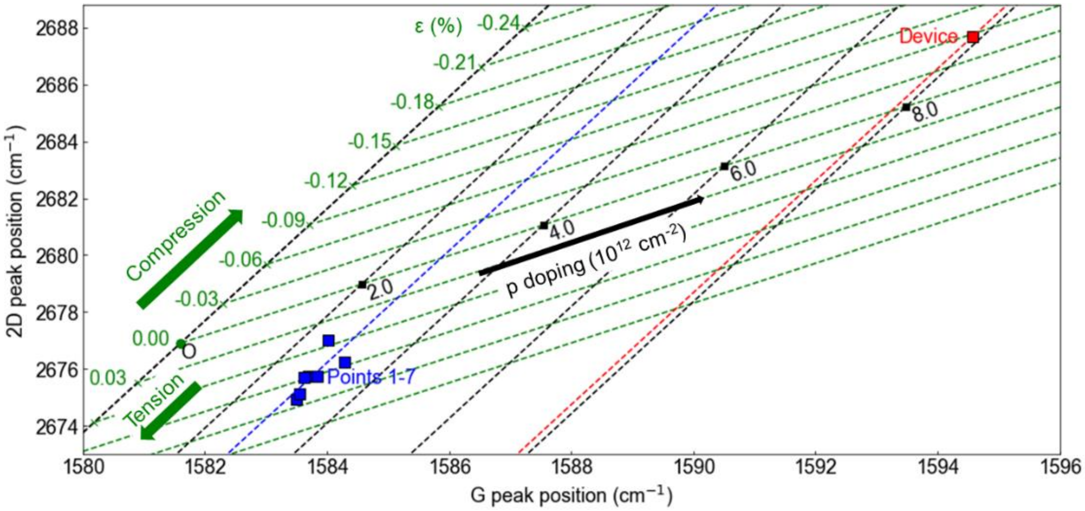

Fig. S2. Peak position of G and 2D. The two group of dashed lines represent the influences from strain (green) and doping (black, blue and red) to graphene. The blue squares are data points of points 1 to 7 with a linear fitted blue dashed line and the red squares are from the device with linear fitted red dashed line.

From the fitted Lorentzians demonstrated in Fig. S1 (b) and (c), the peak positions (pos(2D) and pos(G)), FWHM, and intensity ratio of 2D versus G (I(2D)/I(G)) can be obtained and calculated for doping level calculation according to the dependence curves demonstrated in reference [S2]. Using the relation between FWHM of G peak (11.42 cm^{-1}) and doping level, a concentration of about 1.7×10^{12} cm^{-2} was obtained. And we can verify the doping concentration using three other fitting parameters. From the pos(2D), which is 2687.66 cm^{-1} here, we can tell that the graphene integrated in the device was doped with a concentration of about 5.5×10^{12} cm^{-2}. The results from the relation between concentration and pos(G) (1594.56 cm^{-1}) or the I(2D)/I(G) (1.51) coincide well with the one obtained from the 2D peak information.

Furthermore, we analyzed the strain and strain-induced doping of the graphene on the device according to reference [S3]. The relationship between the peak position of G and 2D are demonstrated in Fig. S2. Firstly, the blue dashed line was obtained by fitting the sample points 1 to 7 with a slope of Δpos(G)/pos(2D) = 1.97 [S3]. And original point O (green solid circle, representing strain-less and doping-neutral condition) is directly adopted from the reference [S3], being (1581.6 ± 0.2, 2676.9 ± 0.7 cm^{-1}). The slope and original point can determine the tensile strain vector (green arrows). Secondly, direction of the doping vector can also be adopted from [S3], with a slope Δpos(2D)/Δpos(G) of 0.7. Afterwards, using the Strain-sensitivity of the G peak

(Δpos(G)/$\Delta\varepsilon$ = -23.5 cm^{-1}/%) and Doping-level-sensitivity of the 2D peak (Δpos(2D)/Δn = 1.04 $cm^{-1}/10^{12}cm^{-2}$) respectively, the corresponding value ranging can be determined. Finally, the strain and doping values of the device can be obtained by plotting the mapping lines, indicating a p-type doping concentration of nearly 8×10^{12} cm^{-2}. And the strain is about -0.06%, demonstrating compressive stress which results from the photoresist cladding.

Section II - Fabrication process flow and morphology of our devices

Device fabrication was based on the SOI platform by multi-project-wafer (MPW) involved processes. Our devices were fabricated on an SOI wafer with a 220-nm-thick device layer, and a 2-µm-thick SiO_2 box layer. Main steps are illustrated in Fig. S3. Step i to step iv were finished by MPW process and the rest fabrication were accomplished in our laboratory center. First, the waveguide structures were patterned by deep ultraviolet photolithography and etched by inductive coupling plasma (ICP) process to a ridge depth of 150 nm and width of 600 nm (step i). Secondly, phosphorus ion implantation was performed to form the n-type lightly doped waveguide areas with a doping concentration of 7×10^{17} cm^{-3} followed by a heavy doping process with a doping concentration of 1.7×10^{20} cm^{-3} for metal contacts (steps iii-iv). Next, a 10-nm-thick dielectric layer of Al_2O_3 was deposited by atomic layer deposition (ALD) as shown in step v. Then the Al_2O_3 windows were opened upon the functional areas of the waveguides and the metal contact areas by chemical etching using a buffered oxide etch (BOE) solution (steps v to vi). The titanium/gold (Ti/Au, 5nm/100nm) contact electrodes were patterned by electron beam lithography (EBL) and deposited using electron beam evaporation (step vii). Then the chemical-vapor-deposition (CVD) graphene on copper substrate was transferred onto the devices by the wet-transfer method[S4] (step viii). Then the loaded graphene was patterned by photolithography, and the unwanted part was etched using oxygen plasma (step ix). Finally, a layer of photoresist protection cladding was coated, and contact windows for characterization were opened by lithography (step x).

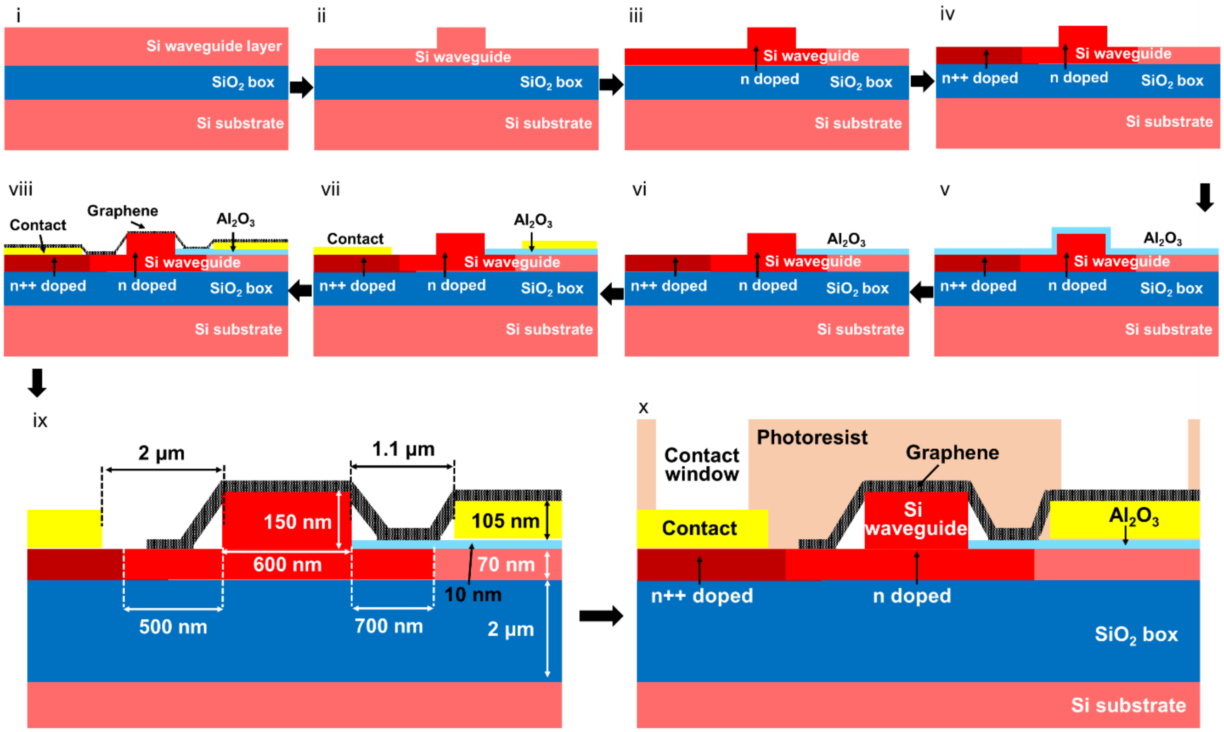

Fig. S3. Fabrication process flow and structural parameters. i: original sample, ii: waveguide fabrication, iii: lightly n-doping of waveguide, iv: heavily n-doping for contact, v: deposition of Al_2O_3, vi: Al_2O_3 window opening, vii: metal contact fabrication, viii: graphene transfer, ix: device after graphene patterning and etching with dimension annotation, x: final device after photoresist coating and patterning. The dimension is not to scale in all steps.

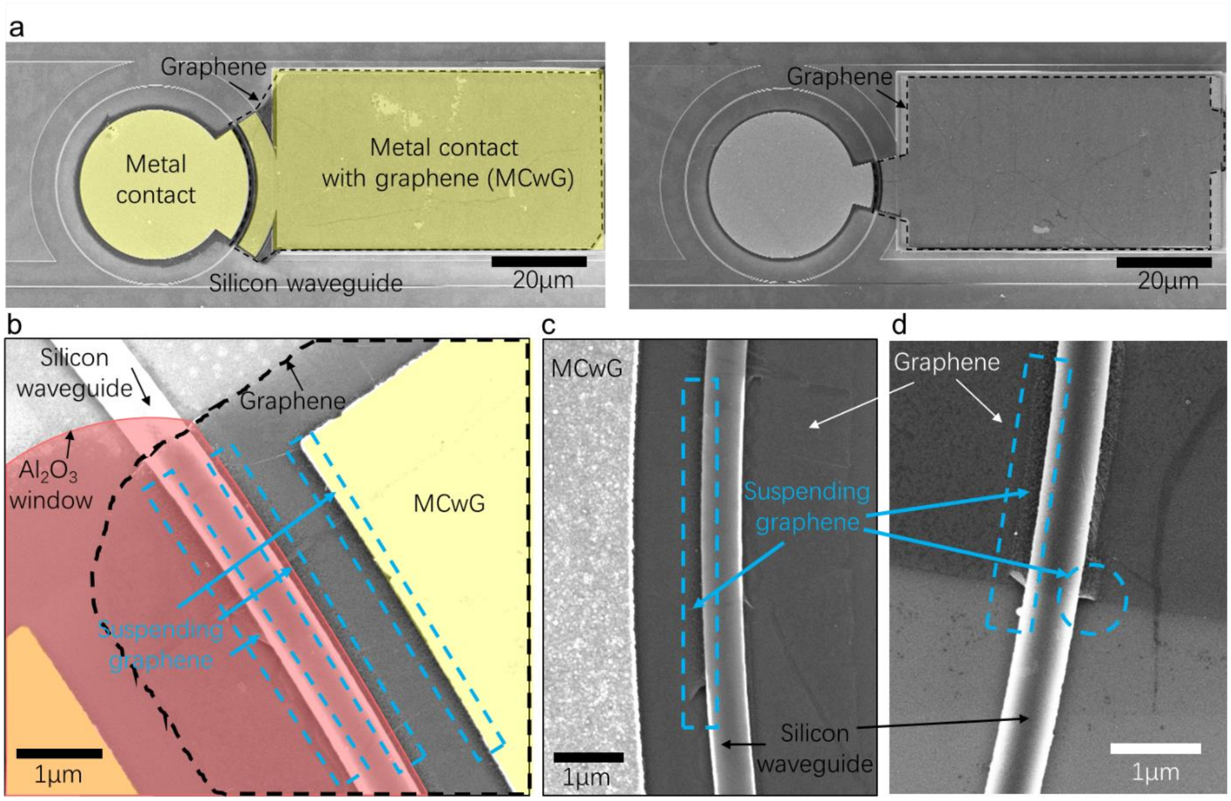

Fig. S4. SEM images of different parts of the devices before photoresist coating. (a)The whole devices with different graphene size. (b) Details of the contact-graphene-waveguide hybrid structure. (c), (d) Details of different parts of the graphene-silicon hybrid structures.

The morphology of our devices are shown in Fig. S4. The integration details of the graphene with other structures is demonstrated in Fig. S4. (b)-(d). It can be clearly observed that the graphene suspended on the waveguide steps (150 nm high) with a good structural integrity.

Section III - Measurement setups

Our device measurement setups are based on a fiber coupling system. The modulator characterization is illustrated in Fig. S5. The computer controlled the wavelength sweeping of the tunable 2 µm laser (Newfocus TLB-6700) and processed the data stream from the data acquisition equipment (DAQ). The polarization of the light was adjusted by a polarization controller to optimize the coupling efficiency. And the transmitted optical signal was read by the photodetector(PD). A source meter (Agilent 2450) was used to apply voltage and monitor the current flowing through the device under test (DUT). The transmission under different voltages can be obtained.

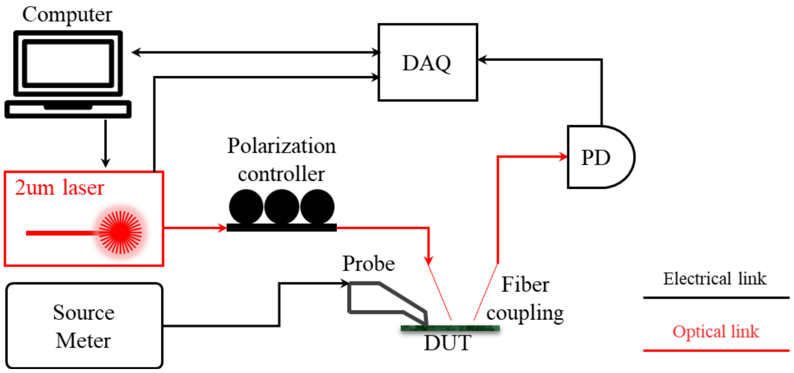

Fig. S5. Schematic measurement setup for modulator characterization. DAQ: data acquisition equipment. PD: photodetector. TDFA: Thulium-doped fiber amplifiers.

The characterization of the detector is illustrated in Fig. S6. For high-power response, the TDFA was introduced. Two optical attenuators were used. The DAQ controlled the optical attenuator 1 to generate power scaling. The optical attenuator 2 was used as a chopper, driven by a signal generator (Siglent SDG6032X-E) with a chopping frequency of 211 Hz, making the optical signal readable for the lock-in amplifier. After the attenuators, the optical signal was split into a reference and major paths. The reference optical signal was measured by a PD and then the two-channel lock-in amplifier (SSI D6104227). And the major signal is measured by the DUT. The current amplifier (SR 570) provided bias voltage and the opto-electric conversion to the DUT, where the photocurrent was amplified and transferred to SSI D6104227. By changing the applied voltage of the optical attenuator 1, the output voltage of the SR 570 and the wavelength of the 2 µm laser, the photocurrent and responsivity under different conditions can be obtained.

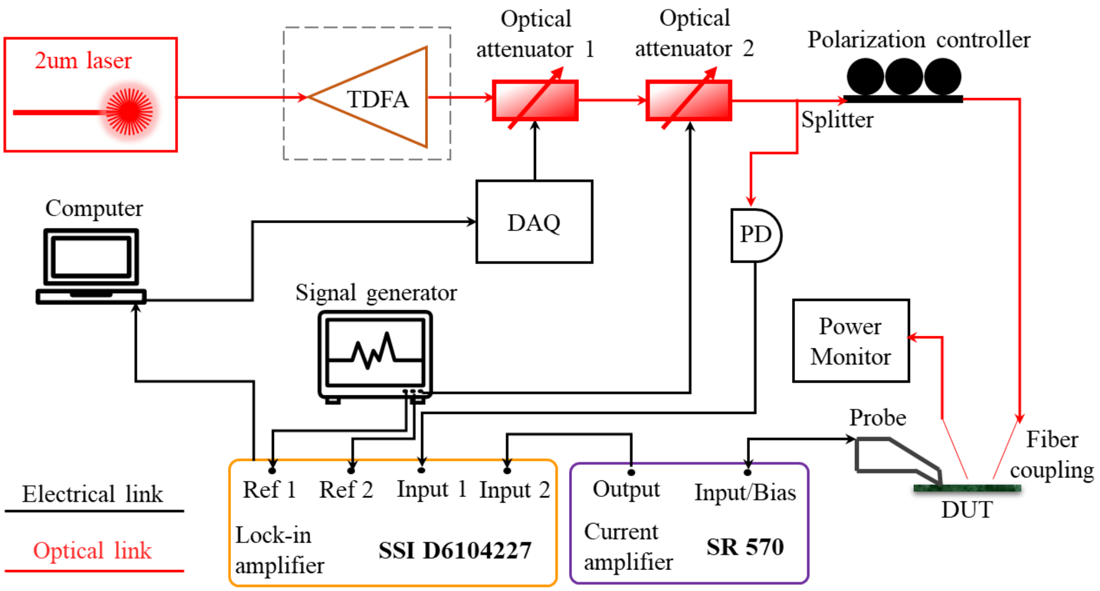

Fig. S6. Schematic of the measurement setup for static characterization of detector.

The activation functions were generated by measuring the output optical signal and the photocurrent of our devices at the same time. The activation function measurement setup (Fig. S7) is based on the photocurrent measurement setup (Fig. S6). In this setup, the output signal through the DUT was converted to photocurrent by a PD and transferred to an additional lock-in amplifier (SR 830) as a power reading. By adding this equipment, we can simultaneously get two-dimensional data diagrams about the photocurrents and output optical power versus bias voltage and input power. And different activation functions can be obtained by mapping photocurrent contours to the optical output diagram. By changing the laser wavelength, we can get various activation functions.

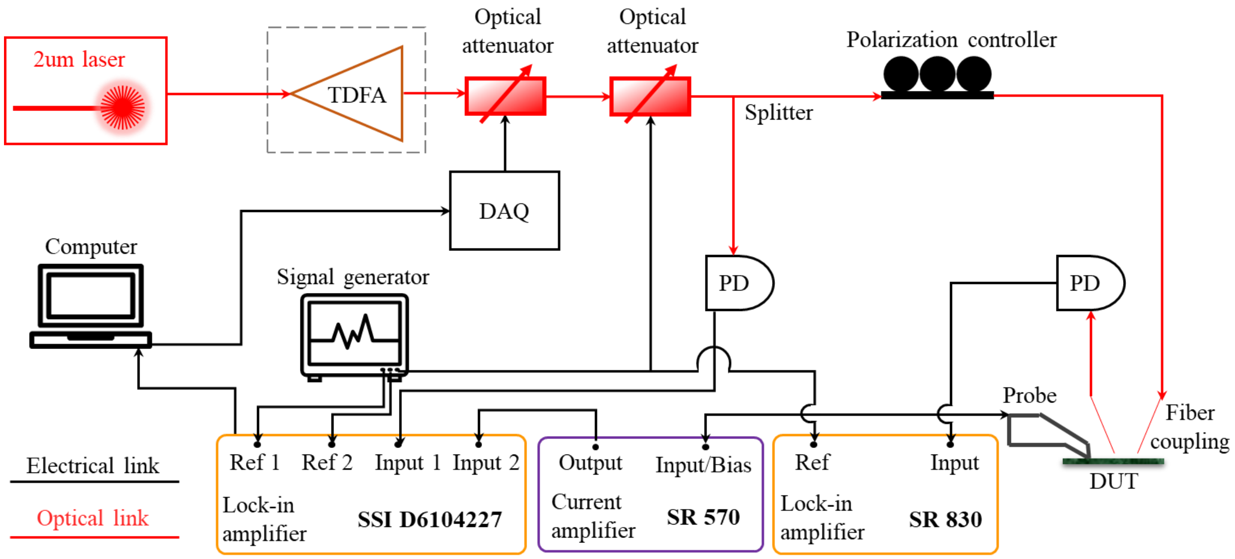

Fig. S7. Schematic measurement setup for the activation function generation.

Section IV - Performance comparison of on-chip graphene-based/silicon-based modulators and detectors

We compare the performances of graphene(Gra)/silicon-based modulators in terms of insertion losses (IL), extinction ratio (ER), and modulation (Mod) power or voltage, and we compare the responsivity and quantum efficiency (QE) for the photodetectors, as shown in Table S2. Our devices nearly excel in all aspects. Among the few modulation-detection-in-one devices, our devices show the smallest footprint, lowest power consumption, highest responsivity, and quantum efficiency.

Table S2. Performance comparison of on-chip silicon/graphene-based modulators and detectors. The 'wg' represents waveguide here. The '-' indicates that the result is not reported in literature and cannot be inferred from data presented.

Device	λ_0 (μm)	Footprint (μm)	Modulation			Photodetection		[Ref]
			IL (dB)	ER (dB)	Mod power or voltage	Responsivity (mA/W)	QE(%)	
Si PIN junction in MRR	1.55	-	< 3	3.0	16.73 mW	-	-	[S5]
Si PIN junction in MZI	1.55	220	0.7	3.26	1.59 mW	-	-	[S6]
Si PIN junction in MRR	1.55	83	3.3	27	2, 8 mW	-	-	[S7]
Si PN junction in MZI	2	2000	15	22	8 V (1.6 V·cm)	-	-	[S8]
Si PIN junction in MZI	2	160	< 2	15.6	10.9 mW	-	-	[S9]
Gra/Al_2O_3 on Si wg	1.55	25	~ 0	~ 2.5	4 V	-	-	[S10]
Gra/Al_2O_3/Gra/ on Si MRR	1.55	5	~ 8	15	50 V	-	-	[S11]
Gra/SiO_2/Si MZI	1.55	300	10	35	7.25 V	-	-	[S12]
Gra/Au slot	1.55	15	6.77-16	-	-	100	8(EQE)	[S13]
Gra/SiO_2 on Si wg	1.55	53	-	-	-	100	3.8 (EQE)	[S14]
Gra/Si junction in Si wg	2.75	150	-	-	-	130	71.5 (IQE)	[S15]
Au/Gra/SiO_2 on Si wg	1.55	24	-	-	-	30	10 (IQE)	[S16]
Gra/Al_2O_3/hBN/ Gra/hBN	1.55	6	-	-	-	500	40 (EQE)	[S17]
Si/Gra-hybrid plasmonic wg	1.55/ 2	50	-	-	-	400/70	32/4.34 (EQE)	[S18]
Si PN junction In MRR	1.55/ 2	750	0.70/ 2.24	15	7.14 mW	140	-	[S19]
Gra/Al_2O_3/Gra FET on Si MZI	1.55	100	-	~ 2	40 V	57	0.25	[S20]
Gra/Si junction in Si MRR	2	20, 50	~ 2	12	0.5 mW (1 V)	200-2000	5-200 (IQE)	This work

Section V - Performance comparison of devices for optical activation function

Table S3. Comparison of state-of-the-art optical activation functions or synapses. The '-' indicates that the result is not reported in literature and cannot be inferred from data presented.

Device	Optical detection feedback	Optical threshold	Electric power or voltage	Footprint (μm^2)	Phase modulation	Reconfi-gurability	[Ref]
**Free-space devices**							
3D-printed SLM	Off-chip	-	0	~2500^2	√	×	[S21]
LC/Si SLM	Off-chip CMOS sensor	-	~17 μW	~9^2	√	√	[S22]
Au/Al_2O_3/Gra SLM with PD * simulation work	Off-chip PD	-	-	60^2	√	√	[S23]
Gra/Ta_2O_5/Gra phototransistor	-	-	>40 V	~30×100	×	-	[S24]
**On-chip all-optical AF devices**							
PCM on Si	-	~2.3 mW	0	~100^2	×	×	[S25]
PCM on Si * free space excitation	-	17 mJ/cm^2	0	-	×	×	[S26]
Gra modulator	-	10 mW	0	~40×10	×	×	[S27]
Ge-Si modulator	Mod-Det in one	1.1 mW	0	~30×8	×	×	[S28]
**On-chip electro-optic AF devices**							
Au/SiO_2/ITO Capacitor	Integrated	5 mW	1.5 mW	~20×0.8	×	√	[S29]
Gra/Al_2O_3/ITO Capacitor	-	~7 mW	~ 20 V	~20×0.5	×	√	[S30]
Si MZIs & MRR TO modulators	-	-	~ 25 mW	~575×48	√	4 AFs	[S31]
Si MZI TO modulator	Off-chip	~60 μW	> 10 V	-	×	4 AFs	[S32]
Si MZI/MRR TO modulators with Ge-Si PD	Integrated	~200 μW	1-2 V	~100^2	×	3 AFs	[S33]
Si MRR TO modulator with Ge-Si PD	Integrated	-	~ 0.9 V	> 90×30	×	-	[S34]
Si MZI & MRR TO modulators with Ge-Si PD	Integrated	-	30 μW	~1000× 100	×	6 AFs	[S35]
Si MZI with ITO/MoS_2/Au opto-resist switch *partially fabricated	Integrated	7 mW/cm^2	2 V	-	×	√	[S36]
Gra/Si heterojunction	Mod-Det in one	~8 μW	0.5 mW/ 1V	~80^2	√	Multi-AFs	This work

Table S3 compare the performance of our Gra/Si heterojunction activation function with other optical activation function devices or synapses. We compare the device prototype, power consumption, operation voltage, functionality, etc. The footprint includes the space which the whole functional photonic device occupies.

The free-space devices include spatial light modulators (SLM), liquid crystal (LC) devices, and graphene (Gra) devices with photodetector (PD) or phototransistor. The footprint here refers to the size of a single pixel of the SLM. Electrically controlled SLMs can achieve phase control and own high reconfigurability but need off-chip PDs or sensors. The on-chip all-optical AF devices include phase change material (PCM) devices and graphene devices. Although no electric power is needed, the optical threshold is usually high. Besides, phase modulation and reconfigurability are not demonstrated.

As for the on-chip electro-optic AF devices, because of the combination of optical modulation (Mod) and detection (Det), high reconfigurability can be achieved. The Indium tin oxide (ITO) film devices exhibited the simplest design, low power consumption of 1.5 mW but high input voltage. In reference [S31] large-footprint design including micro ring resonator (MRR) and Mach-Zehnder interferometer (MZI) circuits were used with high electric power. The most popular approach is called light-splitting-and-detection AF unit [S32, 33, 34, 35, 36, 37], where input optical power is monitored by a PD in an optical bypass, and the photocurrent is transferred to the modulation voltage, feeding back to the transmitted optical power. Our device claims the lowest optical threshold, highest reconfigurability and almost the lowest power consumption, thanks to the modulation-detection-in-one property.

Section VI - Statistical performance characterization of our devices

The modulation and detection results of device 2 with a graphene length of 20 μm are firstly demonstrated in Fig. S8 and Fig. S9, respectively. The graphene length of device 2 is shorter than the one of device 1, resulting in different coupling strengths of the ring resonator. In device 2, the resonance extinction ratio (ER) is deeper under negative bias as shown in Fig. S8b. The same as device 1, the wavelength shift is much more sensitive with voltages larger than 2 V, indicating a thermo-optic effect.

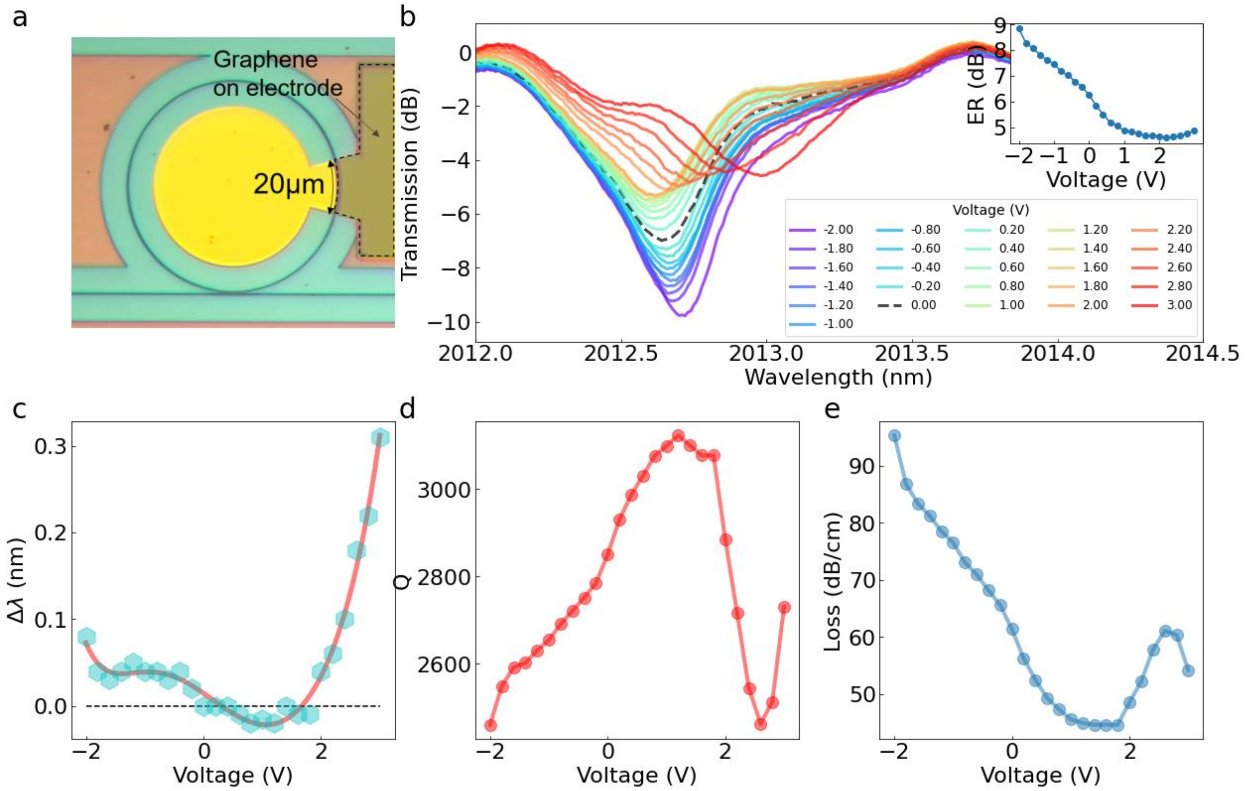

Fig. S8. Characterization of device 2 as a modulator. (a) Microscope image of the device. (b) Transmission under different voltages. The inset shows the resonance extinction ratio (ER) versus voltage. (c) Wavelength shift at different biases. (d) Q factor and (e) calculated loss at different voltages.

The detection measurement results are depicted in Fig. S9. The photocurrent versus bias voltage and input power of the device at four operation wavelengths (2012.57 nm, 2012.60 nm, 2012.61 nm and 2012.69 nm) were measured. The photocurrent is relatively low compared to device 1 because of the shorter graphene, but we think they are acceptable values for an outer electric circuit. And from Fig. S9d, we can also see that the photocurrent became much lower at a voltage larger than 2 V.

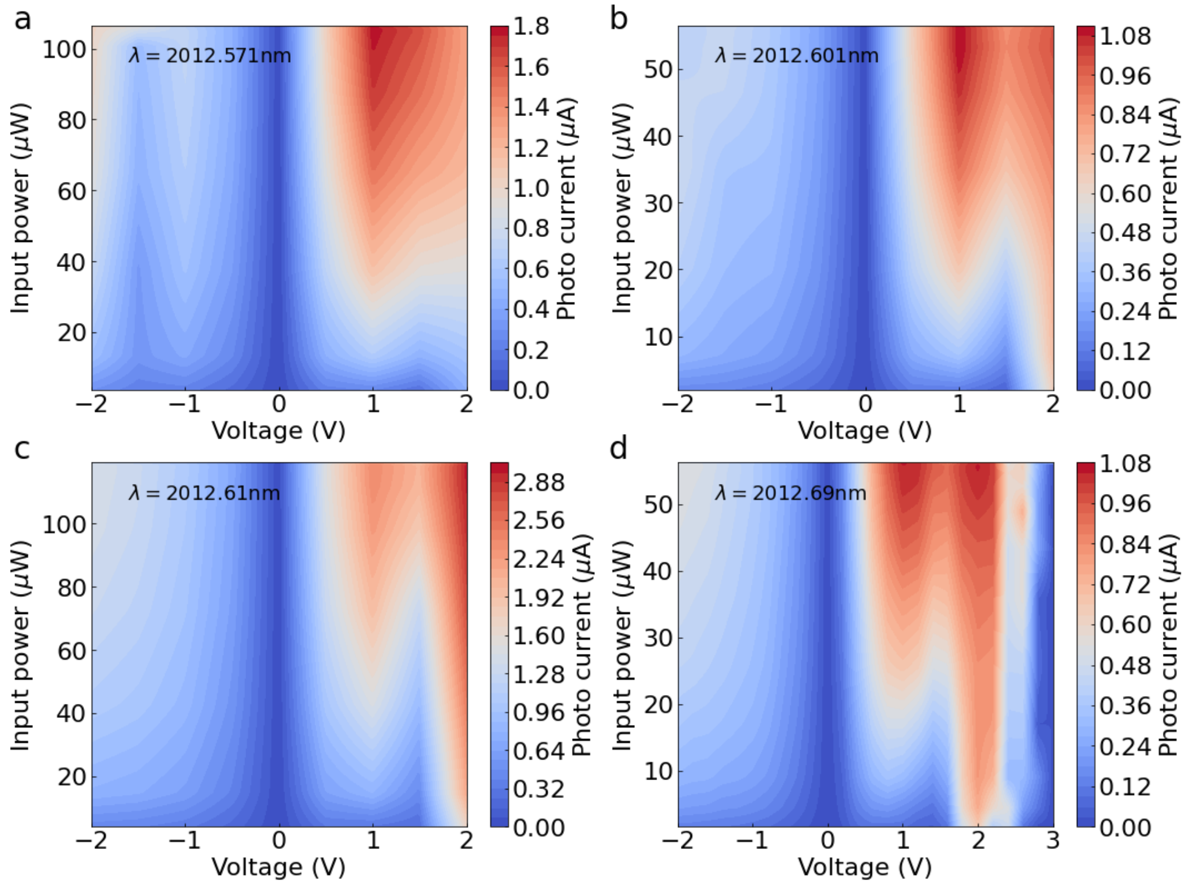

Fig. S9. Characterization of device 2 as a detector at different operation wavelengths.

There are totally 15 heterojunction devices fabricated (in March 2021), which are grouped by the graphene length (L_{gra}: 20, 50 and 100 μm) and ring gap (200 nm to 400 nm). The p-Gra/n-Si heterojunctions were 100% successfully fabricated and the number of devices which can have modulation-detection-in-one functionality is 10/15 (device 1, device 2, devices shown in Fig. S10 and Fig. S11). The spectra of seven devices at different bias voltages as shown in Fig. S10. Due to the varying gap sizes and graphene lengths in different devices, the losses of the micro-rings are different. Hence, the spectra under different voltages of each device are different but exhibit similar evolution tendency compared to device 1's or device 2's. We can predict much more special activation functions that can be generated by all these devices, implying an excellent designability of our device. Last but not least, some of our devices have been newly tested and can still function, showing high reliability for more than two years.

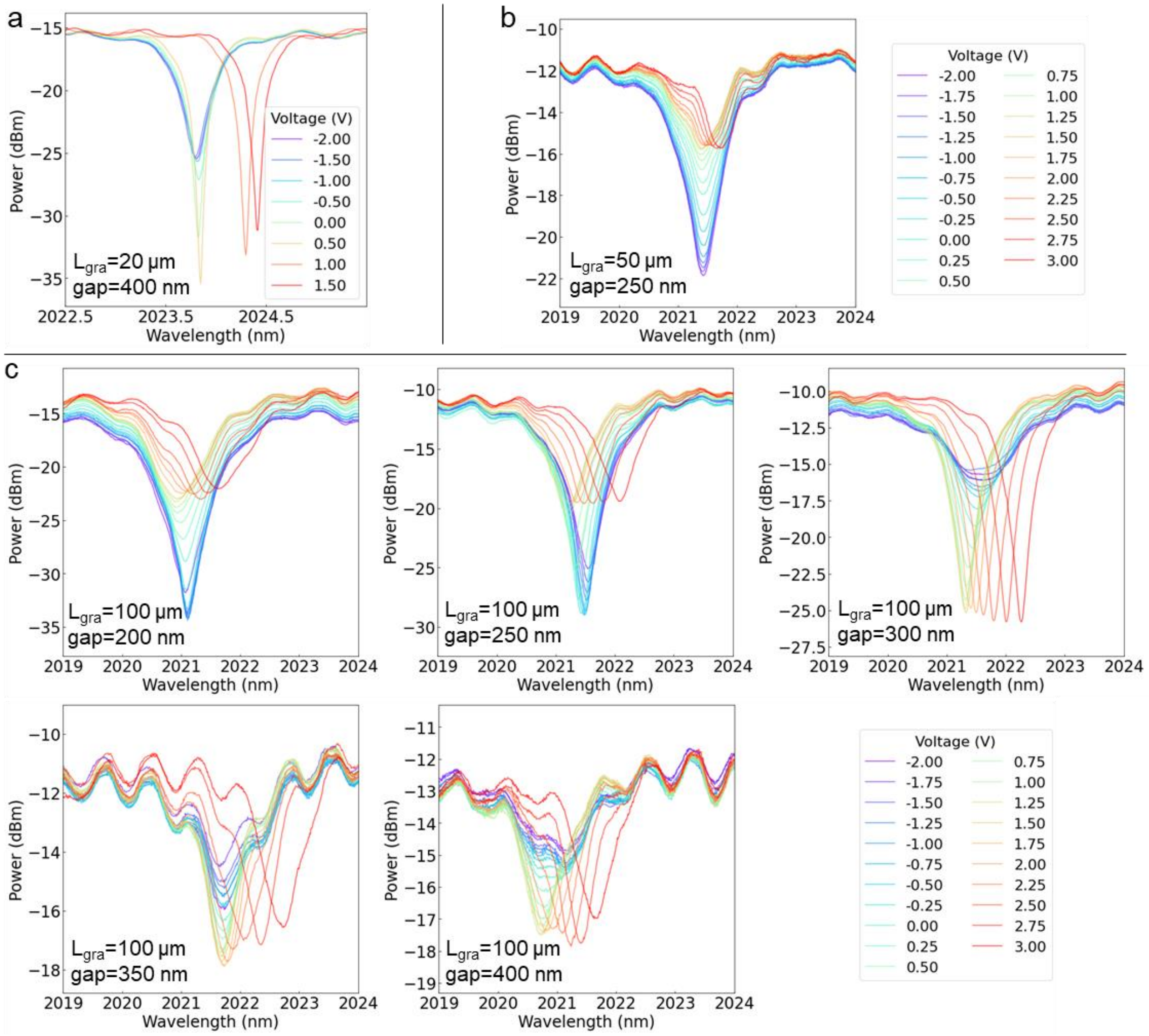

Fig. S10. Spectra of different devices under different voltages. (a)Device with graphene length of 20 μm and ring gap of 400 nm. (b)Device with graphene length of 50 μm and ring gap of 250 nm. (c)Devices with graphene length of 100 μm and ring gaps from 200 nm to 400 nm.

We additionally characterized the modulation and detection performances of a device with L_{gra} of 50 μm and ring gap being 400 nm. The changing trend of the transmission spectrum versus voltages are similar with device 1's, as shown in Fig. S11(a). The I-V curve plotted in Fig. S11(b) verifies the heterojunction feature and the device can achieve a modulation extinction ratio of about 8 dB. The wavelength-resolved responsivity at different voltages under incident optical power of about 0.7 μW were also obtained as shown in Fig. S11(c), where high responsivity were achieved.

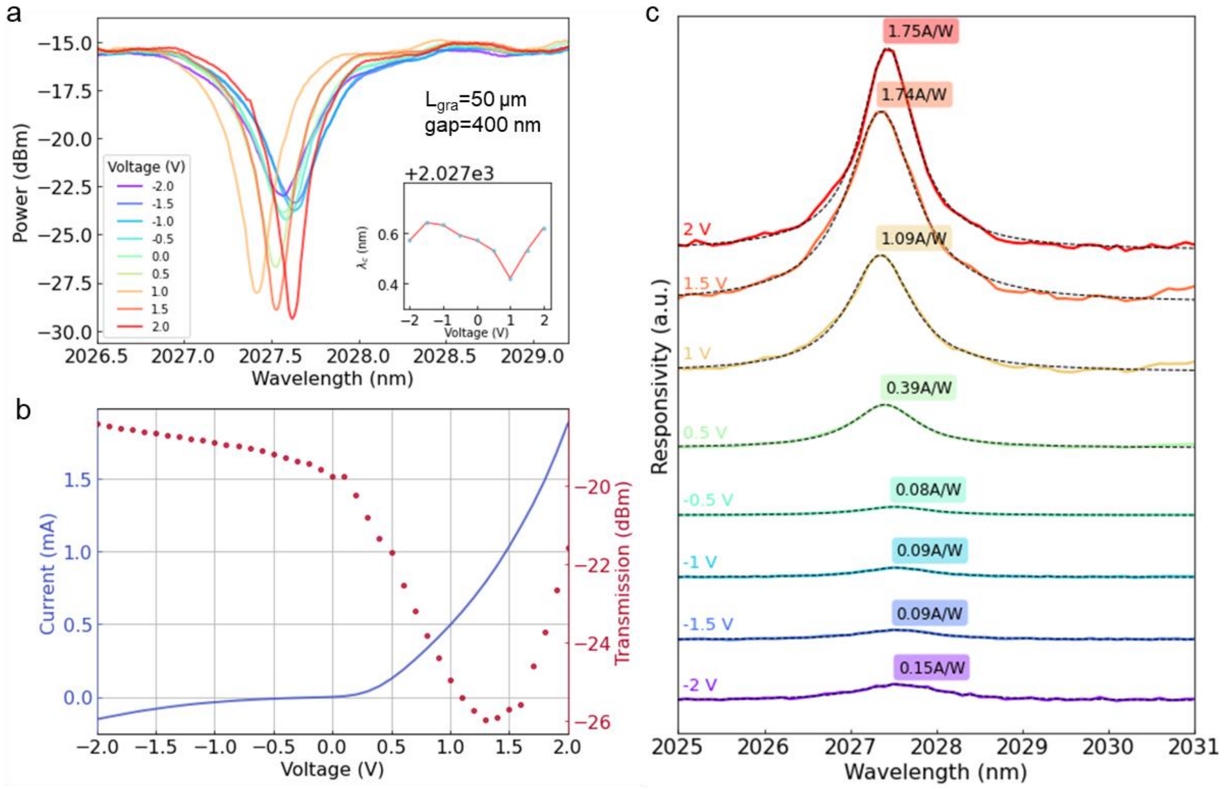

Fig. S11. Performance characterization of device with graphene length of 50 μm and ring gap of 400 nm. (a) Spectra under different voltages. (b) I-V curve (purple line) and transmission variation versus voltage (red dots). (c) Wavelength-resolved responsivity at different voltages under incident optical power of about 0.7 μW.

Section VII - Phase shift deduction of the ring resonator

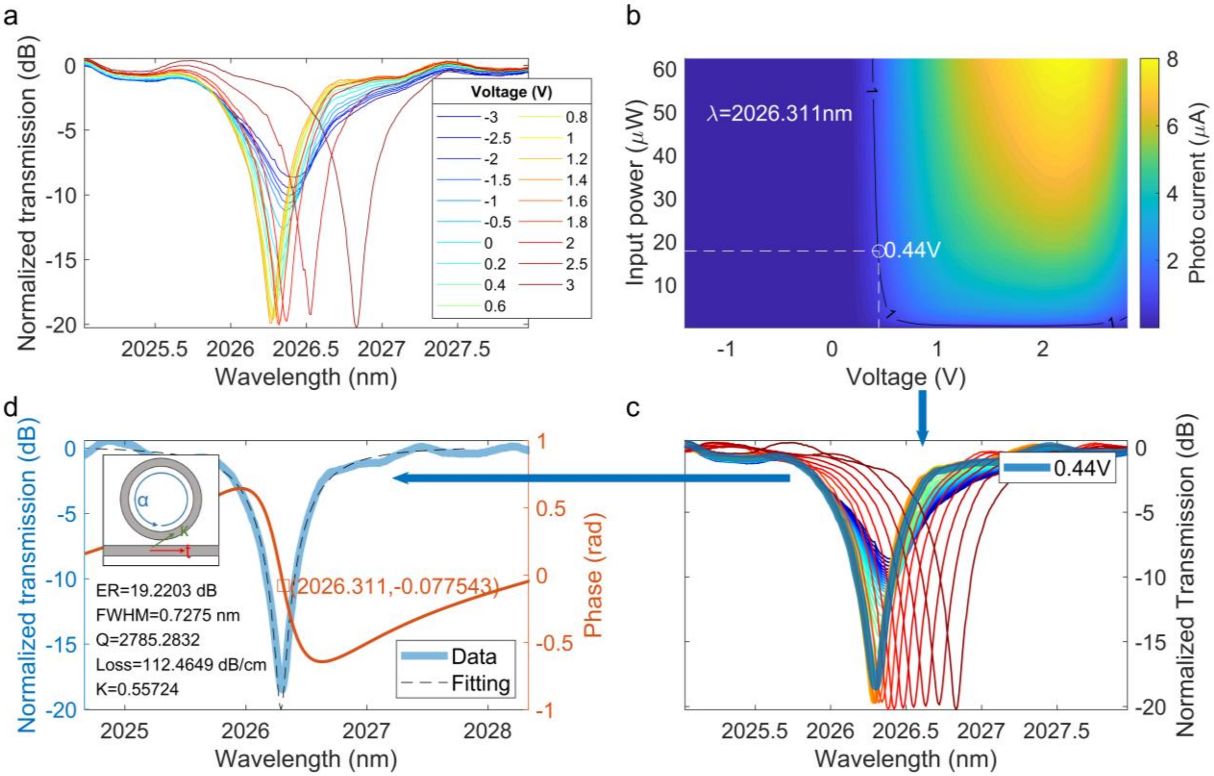

Fig. S12. Example progress of the phase shift deduction at the wavelength of 2026.311 nm. (a) The measured transmission curves. (b) The photocurrent mapping versus voltage and input power at a wavelength of 2026.31 nm. The contour line indicates the photocurrent of 1 μA. (c) Interpolated transmission curves under finer voltage points. The light blue line indicates the transmission at 0.44 V. (d) Transmission at 0.44 V (light blue line) and its fitting curve (dashed line). The orange line is the calculated phase shift. The inset shows the key parameters for the ring resonator calculation. κ: cross-coupling coefficient of the electric field, t: transmit-coupling coefficient of the electric field, α: round-trip loss of the resonator, ER: extinction ratio of the resonance dip, FWHM: full width at a half magnitude of the resonance dip, K: power coupling coefficient of the ring resonator.

The phase shift ϕ of an all-pass single-ring resonator can be calculated from[S38]:

$$\phi = \begin{cases} \tan^{-1}\left[\dfrac{\alpha K \sin(\theta)}{(1+\alpha^2)\sqrt{1-K} - \alpha(2-K)\cos(\theta)}\right], K << 1 \\ \cot^{-1}\left[\dfrac{\alpha K \sin(\theta)}{(1+\alpha^2)\sqrt{K} - \alpha(1+K)\cos(\theta)}\right], 1-K << 1 \end{cases} \qquad \textbf{(IV.1)}$$

where α is the overall amplitude loss in one turn in the resonator, K is the power coupling coefficient of the ring. $\theta = \beta L$ is the round trip phase shift, where $\beta = \dfrac{2\pi n_{eff}}{\lambda}$ is the propagation constant, L is the total length of the ring resonator, with n_{eff} being the effective index of

fundamental mode at wavelength λ. The parameters α and K can be obtained from the transmission curves of the ring resonator.

In our approach, the activation functions are generated from the photocurrent and transmission mapping versus voltage and input power, as described in the main text and demonstrated in Fig. 3. The transmission value in an activation function is related to both the input power and voltage. Therefore, the related voltage should also be verified to obtain the phase shift corresponding to the input power. An example is illustrated in Fig. S12 to show how the phase shift of the device at a wavelength of 2026.311 nm were obtained. Firstly the transmission curves of the device under a limited number of voltages were measured as depicted in Fig. S12a. Then the photocurrent versus input power and voltages of the device at a certain operating wavelength is characterized to extract the photocurrent contour to determine the activation function, where the contour decides the relation between the input power and voltage (Fig. S12b). After the voltage points were extracted, the resonator's transmission curves under those voltages with response to different input power were obtained by the interpolation method as shown in Fig. S12c. The interpolated transmission curves were then fitted by Lorentz function (Fig. S12d) and the parameters α and K of the ring resonator can be calculated using the coupled-wave transfer matrix method[S39]:

$$Q_{loaded} = \frac{\pi L n_g}{\lambda_r |\kappa|^2} = \frac{\lambda_r}{FWHM} \quad \textbf{(IV.2)}$$

$$T = \frac{\alpha^2 + |t|^2 - 2\alpha |t| \cos(\theta)}{1 + \alpha^2 |t|^2 - 2\alpha |t| \cos(\theta)} \quad \textbf{(IV.3)}$$

$$T_{\min} = \frac{(\alpha - |t|)^2}{(1 - \alpha |t|)^2} = 10^{-ER/10} \quad \textbf{(IV.4)}$$

$$n_g = \frac{\lambda_r^2}{FSR \cdot L} \quad \textbf{(IV.5)}$$

$$|\kappa|^2 + |t|^2 = 1 \quad \textbf{(IV.6)}$$

where n_g is the group index. From the transmission (T) curve, the resonance wavelength λ_r, full width at half maximum ($FWHM$) of the resonant dip in linear scale, extinction ratio (ER), free spectral range (FSR) were extracted. Using the equations (IV.2) – (IV.6), the parameters κ, t and α can also calculated, where $|\kappa|^2 = K$. Finally, substituting the calculated values of κ, t and α into the equation (IV.1), the phase shift ϕ of different wavelengths can be calculated as plotted in Fig. S12d.

Section VIII - Details of the optical neural network

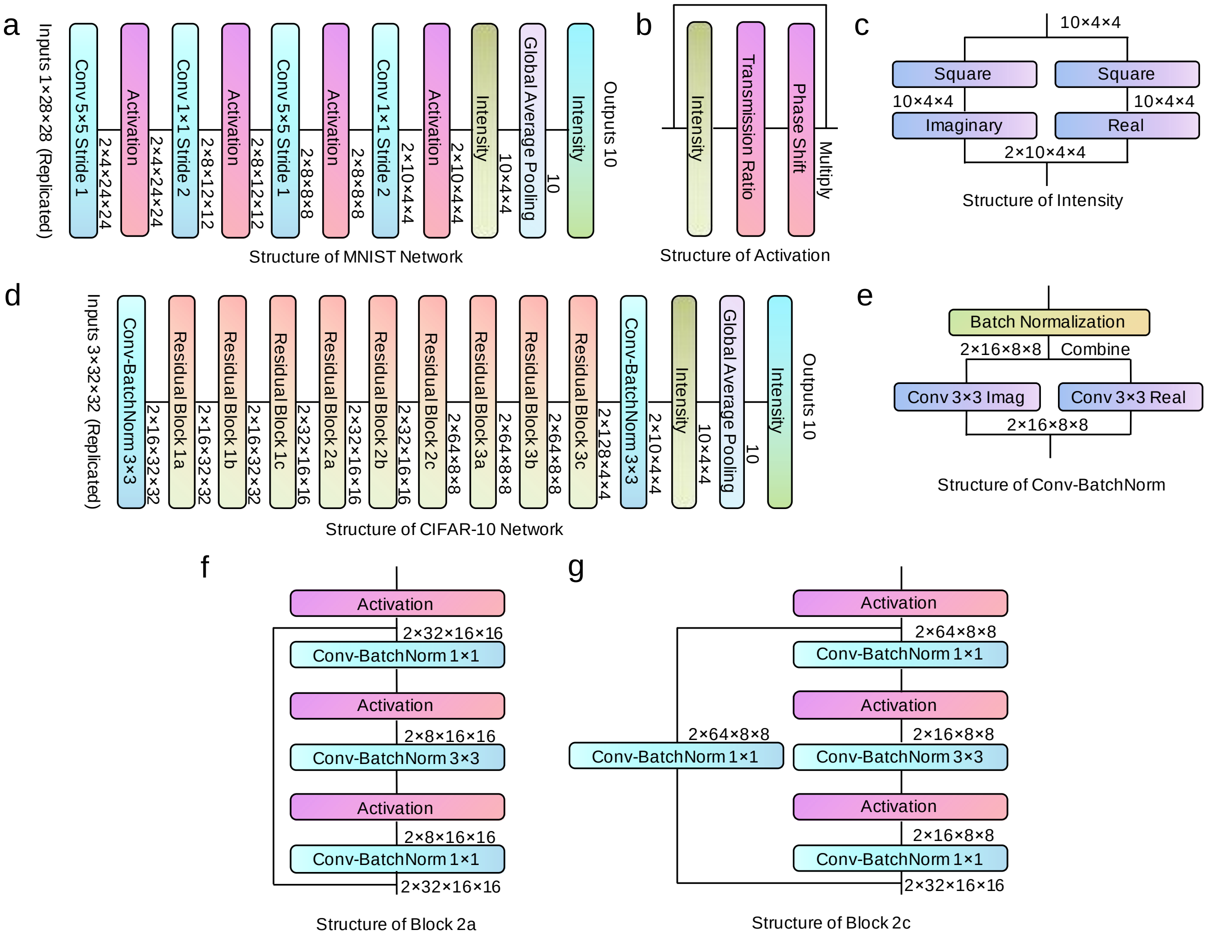

Fig. S13. Schematic diagram of the overall neural network structure used for comparison. (a) Structure diagram of the neural network used for MNIST dataset classification, real values are copied to both real and imaginary channels for input. (b) Structure diagram of the activation layers used in neural networks. This layer applies transmission and phase shift to input complex amplitude using its intensity. (c) Structure diagram of the intensity layers used just before output. (d) Structure diagram of the neural network used for CIFAR-10 dataset classification. (e) Structure diagram of the stacked complex convolution and batch normalization layer used in residual blocks. (f) Structure diagram of the residual block with skip connection and without downsampling, using block 2a as an example. (g) Structure diagram of the residual block with skip connection and strided downsampling, using block 2c as an example.

The two networks based on LeNet and ResNet are shown in Fig. S13a and Fig. S13d. Our designs of the optical networks are based on the original design with similar convolution structures, but we removed the fully connected layers and softmax layers and replaced them with taking the intensity values followed by global average pooling, as the two functions are not compatible with optical hardware. We also replaced the activation layers with the functions obtained by experimental results of our design. The activation layer computes the intensity value with respect to the real and imaginary parts of the complex value. The phase shift and transmission rate can be

obtained from the intensity value, which is used to compute the activated result, as shown in Fig. S13b and Fig. S13c.

Complex values cannot be directly represented with float point numbers, tensor inputs and outputs of complex-valued layers in the network are structured as 2×n×m×m, where n denotes the total number of channels, and m is the size of the feature map. A complex convolution can be performed by applying the real and imaginary parts respectively and combining the results[S40].

The main structure of the network for MNIST dataset (Fig. S13a) is a simple chain of convolution blocks and activation layers, while the network for CIFAR-10 dataset (Fig. S13d) consists of residual blocks with strided downsampling on every third block. Residual blocks are constructed using complex convolution layers with batch normalization applied, as shown in Fig. S13e, and use skip-connections to avoid vanishing gradients for deep networks. The blocks use a stride of 1 for normal blocks and a stride of 2 for downsampling, as shown in Fig. S13f and Fig. S13g**.** Outputs of the final block are then reduced back to 10 channels for global pooling.

For the complex batch normalization layer, we use the naïve approach to apply the real-valued batch normalization function on both the real and imaginary parts of the input. The avoidance of the proper complex batch normalization function[S41] is due to the requirements of the inverse square root of the covariance matrix, which functions as an activation function on the intensity that interferes with our measurement for comparison. After the global pooling, the intensity values of the complex amplitude are normalized using the Softmax function to obtain logits for the final classification results.

For training of the network, we use ADAM optimizer[S42] with $\beta_1 = 0.9, \beta_2 = 0.999$, and adopt the weight initialization[S43] with no dropout. These models are trained with a minibatch size of 64 on a single NVIDIA RTX 3090 GPU. We start with a learning rate of 0.001 and terminate training at 10 rounds for MNIST and 40 rounds for CIFAR-10 using the cross-entropy loss function. We apply no data augmentation to focus on the task of evaluating different activation functions. For each activation function, the model is randomly initialized and trained for 5 times. A typical training process takes up around 1 hour.

Section IX - Common activation functions and our optical activation functions used in training

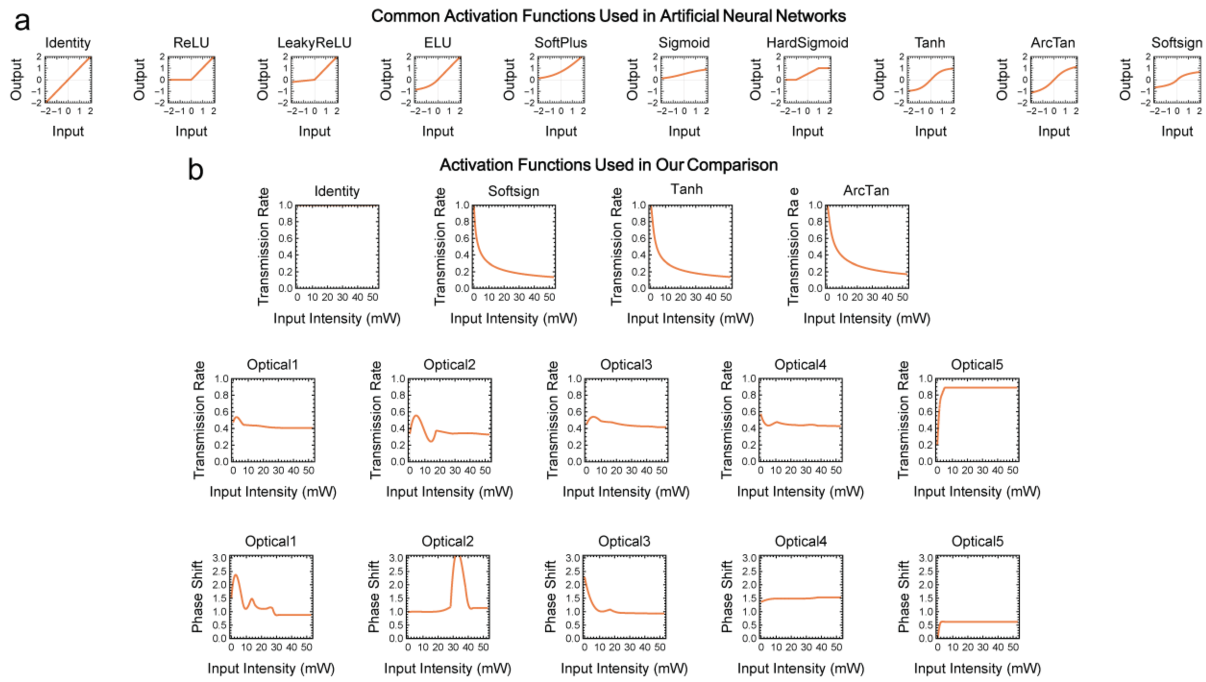

Fig. S14. Properties of activation functions. (a) Examples of commonly used activation functions adapted in real-valued neural networks. (b) Transmission rate and relative phase shift curves of the activation functions used in our network training.

We choose some classical activation functions in real-valued neural networks (Tanh, Arctan and Softsign functions) for the comparison due to the fact that a significant portion of activation functions used are not applicable for complex-valued optical neural networks that perform activation on intensity. Most activation functions are not symmetrical over positive and negative values, i.e., $f(x) \neq -f(-x)$, which is incompatible with positive-only intensity values. The frequently used ReLU, $f(x) = \max(0, x)$, will collapse to $f(x) = x$ when $x>0$, identical to using no activation at all. The same applies to many variants of the ReLU function including the Leaky ReLU, ELU functions[S44, 45]. For the Softplus function, Sigmoid function and its variant Hard Sigmoid[S46], $f(x) \neq 0$ when $x=0$, they do not conform to the characteristics of complex-valued optical neural networks that perform activation on intensity as well, leaving only odd functions such as Tanh, Arctan and Softsign available for usage.

Commonly used activations in neural networks are shown in Fig. S14a, which contains Identity for reference; ReLU function and its variant Leaky ReLU, ELU functions, where $f(x)$ is asymmetrical over the domain; Softplus function; Sigmoid function and its variant Hard Sigmoid, where $f(x) \neq 0$ when $x=0$; Odd functions including Tanh, Arctan and Softsign.

In Fig. S14b, both the transmission and relative phase shift curves are demonstrated. The identity function and the common activation functions are assumed to have a phase shift of 0. Optical activation functions are interpolated and extrapolated to cover the domain of values in the neural

network, allowing for backward propagation gradient computation. The classical activation functions are assumed to have a phase shift of 0.

Section X - Details of the optical activation functions

For implementing the simulated optical neural network, we use Apache MXNet, an open-source deep learning software framework, which is integrated with Wolfram Mathematica, allowing for flexible network training and visualization.

The MXNet does not support complex numbers and complex operations, so our implementation wraps upon the original 2D convolution function, using four separate convolutions on the real and imaginary part of the input with the real and imaginary weights, concatenating the results afterward.

For the activation functions, as the data comes in discrete points that are not continuous or differentiable, an interpolation has to be made to allow for network training. However, the points are more than necessary to get a well-fit interpolated piecewise function, and a piecewise function with too many points will slow down neural network training by a significant portion, thus, some optimization is required. Prior to the final output, we did a third-order spline interpolation on all of the points, and as the function is now differentiable, we picked out all the points that are a turning point (local maxima/minima of second-order derivative), which is used with third order spline interpolation to get the final interpolated function.

The interpolated functions of the intensity and phase functions reflect the transmission ratio on intensity and the phase shift with respect to input intensity. To apply the function on the complex amplitude, we take 1 minus the square root of the transmission values as the multiplier ratio. For input complex amplitude, we compute the intensity values being the sum of squares of the real and imaginary parts and use this value on the interpolated function to get the ratio, multiplying it to the two parts. After this, we transform the complex amplitude to intensity-phase expression and apply the phase shift, then convert it back to real and imaginary parts. The expression of the five optical activation functions, including the ratio and phase shift part, are shown below.

Transmission rate interpolation functions:

Optical activation function 1

$$\begin{cases}
(x-0.384466)((0.0012644(x-7.07702)-0.00906869)(x+0.)+0.0563937)+0.495598 & x<0.384466 \\
(x-7.07702)((x-0.384466)(0.0012644(x+0.)-0.00906869)-0.00778564)+0.443492 & x<7.07702 \\
((0.0000218753(x-11.8108)-0.0000854345)(x-7.07702)-0.000942025)(x-7.89319)+0.442723 & x<7.89319 \\
((0.0000218753(x-7.07702)-0.0000854345)(x-7.89319)-0.00134645)(x-11.8108)+0.437448 & x<11.8108 \\
\left((9.01582\times10^{-6}(x-15.4019)+0.000125094)(x-11.8108)-0.00101854\right)(x-13.6063)+0.43562 & x<13.6063 \\
(x-15.4019)((x-13.6063)(0.000125094-0.0000388262(x-11.8108))-0.000569308)+0.434597 & x<15.4019 \\
((0.0000234554(x-13.6063)-0.000236156)(x-15.4019)-0.00234254)(x-21.1151)+0.421214 & x<21.1151 \\
((0.0000129116(x-27.4812)+0.000153358)(x-21.1151)-0.0023638)(x-23.5636)+0.415426 & x<23.5636 \\
(x-27.4812)((x-23.5636)(0.0000885621-7.78337\times10^{-6}(x-29.4399))-0.00138751)+0.409991 & x<27.4812 \\
(x-29.4399)((x-27.4812)(0.0000829955-6.70092\times10^{-7}(x-31.2355))-0.000867083)+0.408292 & x<29.4399 \\
(x-31.2355)((x-29.4399)(0.0000793859-6.70092\times10^{-7}(x-32.8678))-0.000555488)+0.407295 & x<31.2355 \\
(x-32.8678)((x-31.2355)(0.000023541-0.0000106912(x-34.6634))-0.000283362)+0.406832 & x<32.8678 \\
0.406832 & \text{True}
\end{cases}$$

Optical activation function 2

$$\begin{cases}
(x-1.36386)((0.000658985(x-9.36228)-0.0109557)(x+0.)+0.0931759)+0.463102 & x<1.36386 \\
(x-9.36228)((x-1.36386)(0.000658985(x+0.)-0.0109557)-0.00939405)+0.387965 & x<9.36228 \\
((0.000658985(x-1.36386)+0.000484787)(x-9.36228)-0.00163899)(x-17.3607)+0.374855 & x<17.3607 \\
\left((9.54288\times10^{-6}(x-9.36228)-0.0000928324)(x-17.3607)-0.00339677\right)(x-28.2973)+0.337706 & x<28.2973 \\
\left((3.09805\times10^{-6}(x-41.1927)-0.0000519236)(x-28.2973)+0.00042435\right)(x-38.2545)+0.341932 & x<38.2545 \\
\left((-0.0000374278(x-42.9883)-6.41017\times10^{-6})(x-38.2545)-0.000245225\right)(x-41.1927)+0.341211 & x<41.1927 \\
\left((-0.0000374278(x-38.2545)-6.41017\times10^{-6})(x-41.1927)-0.00027557\right)(x-42.9883)+0.340716 & x<42.9883 \\
((0.0000535052(x-47.722)+0.0000496883)(x-42.9883)-0.00174302)(x-46.0897)+0.33531 & x<46.0897 \\
(x-47.722)\left((x-46.0897)\left(0.0000141538-5.30957\times10^{-6}(x-49.6808)\right)-0.00150781\right)+0.332849 & x<47.722 \\
\left((2.64324\times10^{-6}(x-46.0897)+0.0000141538)(x-47.722)-0.00145698\right)(x-49.6808)+0.329995 & x<49.6808 \\
((0.0000186375(x-47.722)+0.0000296865)(x-49.6808)-0.00133099)(x-51.9661)+0.326954 & x<51.9661 \\
0.326954 & \text{True}
\end{cases}$$

Optical activation function 3

$$
\begin{cases}
(x-3.81236)((0.000227083(x-9.52552)-0.00339264)(x+0.)+0.0239639)+0.537748 & x<3.81236 \\
(x-9.52552)((x-3.81236)(0.000227083(x+0.)-0.00339264)-0.0083527)+0.490028 & x<9.52552 \\
(x-17.0342)((x-9.52552)(0.000475547-0.0000302601(x-3.81236))-0.00206508)+0.474522 & x<17.0342 \\
(x-24.0533)((x-17.0342)(0.00015939-2.83564\times 10^{-6}(x-28.1341))-0.00405457)+0.446063 & x<24.0533 \\
(x-28.1341)((x-24.0533)(0.000122823-9.46824\times 10^{-6}(x-29.9296))-0.00228536)+0.436736 & x<28.1341 \\
(x-29.9296)((x-28.1341)(0.0000455469-9.46824\times 10^{-6}(x-32.2149))-0.0015636)+0.433929 & x<29.9296 \\
(x-32.2149)((x-29.9296)(0.0000455469-3.35855\times 10^{-6}(x-28.1341))-0.00137774)+0.43078 & x<32.2149 \\
(x-34.5002)((x-32.2149)(0.0000241661-3.35855\times 10^{-6}(x-29.9296))-0.00126728)+0.427884 & x<34.5002 \\
(x-39.0707)((x-34.5002)(0.000102735-8.44547\times 10^{-6}(x-32.2149))-0.000562956)+0.425311 & x<39.0707 \\
((-0.0000129561(x-47.722)-0.000160933)(x-39.0707)-0.000473242)(x-43.1515)+0.42338 & x<43.1515 \\
((0.0000163902(x-51.6396)+0.0000450737)(x-43.1515)-0.00186553)(x-47.722)+0.414854 & x<47.722 \\
0.414854 & \text{True}
\end{cases}
$$

Optical activation function 4

$$
\begin{cases}
(x-1.82984)((x+0.)(0.00436266-0.000366951(x-10.9054))-0.0485221)+0.483716 & x<1.82984 \\
(x-10.9054)((x-1.82984)(0.00436266-0.000366951(x+0.))-0.000945582)+0.475134 & x<10.9054 \\
(x-13.1189)((x-10.9054)(0.000441305-0.0000248121(x-15.5538))-0.0060409)+0.461762 & x<13.1189 \\
(x-15.5538)((x-13.1189)(0.000441305-0.0000248121(x-10.9054))-0.00398951)+0.452048 & x<15.5538 \\
(x-18.2101)((x-15.5538)(0.00026006-4.67123\times 10^{-6}(x-13.1189))-0.0026655)+0.444968 & x<18.2101 \\
(x-22.8585)((x-18.2101)(0.000214564-0.0000154797(x-15.5538))-0.00109818)+0.439863 & x<22.8585 \\
((0.0000395341(x-28.6138)+0.000433979)(x-22.8585)-0.000877294)(x-27.9497)+0.435397 & x<27.9497 \\
(x-28.6138)((x-27.9497)(0.000433979-0.0000448605(x-22.8585))+0.00162035)+0.436473 & x<28.6138 \\
((-0.0000448605(x-27.9497)-0.000231337)(x-28.6138)-0.000632782)(x-37.6893)+0.43073 & x<37.6893 \\
\left((-1.4592\times 10^{-6}(x-54.2909)-0.0000138405)(x-37.6893)-0.000193453\right)(x-47.2076)+0.428888 & x<47.2076 \\
(x-54.2909)((x-47.2076)(0.0000141043-4.17588\times 10^{-6}(x-57.6113))-0.000423227)+0.425891 & x<54.2909 \\
\left((-4.17588\times 10^{-6}(x-60.7102)-0.0000422811)(x-54.2909)-0.000276491\right)(x-57.6113)+0.424972 & x<57.6113 \\
0.424972 & \text{True}
\end{cases}
$$

Optical activation function 5

$$
\begin{cases}
(x-1.38713)((-0.0224503(x-1.60848)-0.0649532)(x+0.)+0.312567)+0.640487 & x<1.38713 \\
((-0.0288155(x-1.82984)-0.106034)(x-1.38713)+0.208091)(x-1.60848)+0.686549 & x<1.60848 \\
((-0.0288155(x-2.05119)-0.125169)(x-1.60848)+0.161149)(x-1.82984)+0.72222 & x<1.82984 \\
((0.13057(x-2.27255)-0.038462)(x-1.82984)+0.105735)(x-2.05119)+0.745625 & x<2.05119 \\
((0.0108098(x-2.4939)-0.0312836)(x-2.05119)+0.088708)(x-2.27255)+0.765261 & x<2.27255 \\
((-0.0162407(x-2.05119)-0.0312836)(x-2.27255)+0.0748585)(x-2.4939)+0.781831 & x<2.4939 \\
((0.0585022(x-3.15797)+0.00613563)(x-2.4939)+0.044535)(x-2.93661)+0.801547 & x<2.93661 \\
((0.00139523(x-3.37932)+0.00737099)(x-2.93661)+0.0486095)(x-3.15797)+0.812307 & x<3.15797 \\
((0.00212891(x-3.60068)+0.00878473)(x-3.15797)+0.0518727)(x-3.37932)+0.823789 & x<3.37932 \\
((0.00212891(x-3.15797)+0.00878473)(x-3.37932)+0.0557618)(x-3.60068)+0.836132 & x<3.60068 \\
((0.0269896(x-4.92881)+0.036337)(x-3.60068)+0.0484797)(x-4.70745)+0.889788 & x<4.70745 \\
0.889788 & \text{True}
\end{cases}
$$

Phase interpolation functions:

Optical activation function 1

$$
\begin{cases}
\left((0.00774108(x-7.89319)-0.0308937)(x-0.384466)-0.0380957\right)(x-7.07702)+1.48205 & x<7.07702 \\
(x-7.89319)((x-7.07702)(0.0575584-0.00569566(x-11.8108))-0.270068)+1.26163 & x<7.89319 \\
x-11.8108)((x-7.89319)(0.0575584-0.00569566(x-7.07702))+0.00239964)+1.27103 & x<11.8108 \\
((-0.0114138(x-15.4019)-0.0653337)(x-11.8108)+0.118774)(x-13.6063)+1.4843 & x<13.6063 \\
((0.00826121(x-11.8108)-0.0653337)(x-13.6063)-0.115847)(x-15.4019)+1.27629 & x<15.4019 \\
(x-21.1151)((x-15.4019)(0.011531-0.000782363(x-13.6063))-0.0292644)+1.1091 & x<21.1151 \\
((-0.000314609(x-27.4812)-0.0000593992)(x-21.1151)+0.001267)(x-23.5636)+1.1122 & x<23.5636 \\
((-0.002477(x-29.4399)-0.0206802)(x-23.5636)+0.000888856)(x-27.4812)+1.11568 & x<27.4812 \\
(x-29.4399)((x-27.4812)(0.032003-0.00595078(x-31.2355))-0.120636)+0.879379 & x<29.4399 \\
((-0.00595078(x-32.8678)-0.000051969)(x-29.4399)-0.000484958)(x-31.2355)+0.878508 & x<31.2355 \\
((6.76304\times 10^{-6}(x-34.6634)-0.0000166425)(x-31.2355)-0.000663102)(x-32.8678)+0.877426 & x<32.8678 \\
0.877426 & \text{True}
\end{cases}
$$

Optical activation function 2

$$\begin{cases} ((0.0000308766(x-17.3607)+0.0000105626)(x-1.36386)+0.000219814)(x-9.36228)+0.995515 & x<9.36228 \\ ((0.0000308766(x-1.36386)+0.0000105626)(x-9.36228)+0.000388781)(x-17.3607)+0.998625 & x<17.3607 \\ ((0.0000622563(x-9.36228)+0.000842175)(x-17.3607)+0.0163354)(x-28.2973)+1.17728 & x<28.2973 \\ ((0.00545092(x-41.1927)-0.0255344)(x-28.2973)+0.0715139)(x-38.2545)+1.88936 & x<38.2545 \\ (x-41.1927)((x-38.2545)(0.0545449-0.00695953(x-42.9883))-0.257762)+1.132 & x<41.1927 \\ (x-42.9883)((x-41.1927)(0.0545449-0.00695953(x-38.2545))+0.000440333)+1.13279 & x<42.9883 \\ \left((-3.01628\times10^{-6}(x-47.722)-4.00111\times10^{-6})(x-42.9883)+0.000517182\right)(x-46.0897)+1.1344 & x<46.0897 \\ \left((-2.69823\times10^{-6}(x-49.6808)-0.0000220591)(x-46.0897)+0.000498242\right)(x-47.722)+1.13521 & x<47.722 \\ \left((1.48452\times10^{-6}(x-46.0897)-0.0000220591)(x-47.722)+0.000419025\right)(x-49.6808)+1.13603 & x<49.6808 \\ \left((6.80467\times10^{-7}(x-47.722)-0.0000133355)(x-49.6808)+0.000362428\right)(x-51.9661)+1.13686 & x<51.9661 \\ 1.13686 & \text{True} \end{cases}$$

Optical activation function 3

$$\begin{cases} (x-9.52552)((x-3.81236)(0.00664162-0.000413152(x-17.0342))-0.0808787)+1.02912 & x<9.52552 \\ (x-17.0342)((x-9.52552)(0.00664162-0.000413152(x-3.81236))+0.00693591)+1.0812 & x<17.0342 \\ (x-24.0533)((x-17.0342)(0.00150162-0.000111753(x-28.1341))-0.0180656)+0.954393 & x<24.0533 \\ (x-28.1341)((x-24.0533)(0.0000605263-9.25655\times10^{-6}(x-29.9296))-0.00139785)+0.948689 & x<28.1341 \\ \left((-9.25655\times10^{-6}(x-32.2149)-0.0000150224)(x-28.1341)-0.00104217\right)(x-29.9296)+0.946817 & x<29.9296 \\ ((-0.0000127964(x-28.1341)-0.0000150224)(x-29.9296)-0.00110348)(x-32.2149)+0.944296 & x<32.2149 \\ ((-0.0000127964(x-29.9296)-0.0000964855)(x-32.2149)-0.00154447)(x-34.5002)+0.940766 & x<34.5002 \\ (x-39.0707)((x-34.5002)(0.000113688-0.0000112807(x-32.2149))-0.000765046)+0.93727 & x<39.0707 \\ \left((1.5899\times10^{-6}(x-47.722)+0.0000113362)(x-39.0707)-0.000848836\right)(x-43.1515)+0.933806 & x<43.1515 \\ \left((-1.99716\times10^{-6}(x-51.6396)-0.000013766)(x-43.1515)-0.000750763\right)(x-47.72)+0.930374 & x<47.722 \\ 0.930374 & \text{True} \end{cases}$$

Optical activation function 4

$$\begin{cases} ((0.000057092(x-13.1189)-0.000784946)(x-1.82984)+0.00895295)(x-10.9054)+1.48838 & x<10.9054 \\ \left((-4.87929\times10^{-8}(x-15.5538)-1.41511\times10^{-6})(x-10.9054)+0.0000916229\right)(x-13.1189)+1.48858 & x<13.1189 \\ \left((-4.87929\times10^{-8}(x-10.9054)-1.41511\times10^{-6})(x-13.1189)+0.0000850448\right)(x-15.5538)+1.48879 & x<15.5538 \\ \left((-2.79728\times10^{-7}(x-13.1189)-1.77153\times10^{-6})(x-15.5538)+0.0000760257\right)(x-18.2101)+1.48899 & x<18.2101 \\ \left((5.0112\times10^{-9}(x-15.5538)-4.49597\times10^{-6})(x-18.2101)+0.000043184\right)(x-22.8585)+1.48919 & x<22.8585 \\ \left((5.44478\times10^{-6}(x-28.6138)+0.0000522119)(x-22.8585)+0.\right)(x-27.9497)+1.48919 & x<27.9497 \\ ((0.0000274283(x-22.8585)+0.0000522119)(x-27.9497)+0.000300491)(x-28.6138)+1.48939 & x<28.6138 \\ ((0.0000274283(x-27.9497)+0.000458994)(x-28.6138)+0.00477092)(x-37.6893)+1.53269 & x<37.6893 \\ \left((0.0000100417(x-54.2909)+6.63693\times10^{-7})(x-37.6893)-0.0000110184\right)(x-47.2076)+1.53259 & x<47.2076 \\ \left((6.13152\times10^{-7}(x-57.6113)-3.15916\times10^{-6})(x-47.2076)+0.\right)(x-54.2909)+1.53259 & x<54.2909 \\ \left((6.13152\times10^{-7}(x-60.7102)+5.12002\times10^{-6})(x-54.2909)-0.0000328669\right)(x-57.6113)+1.53248 & x<57.6113 \\ 1.53248 & \text{True} \end{cases}$$

Optical activation function 5

$$\begin{cases} ((-0.0250725(x-1.82984)-0.14196)(x-1.38713)+0.192884)(x-1.60848)+0.565263 & x<1.60848 \\ ((-0.0250725(x-2.05119)-0.15861)(x-1.60848)+0.130037)(x-1.82984)+0.594048 & x<1.82984 \\ ((0.174222(x-2.27255)-0.0429153)(x-1.82984)+0.0598185)(x-2.05119)+0.607289 & x<2.05119 \\ ((0.0141581(x-2.4939)-0.0335134)(x-2.05119)+0.0408195)(x-2.27255)+0.616324 & x<2.27255 \\ ((0.00618108(x-2.05119)-0.0335134)(x-2.27255)+0.0259828)(x-2.4939)+0.622076 & x<2.4939 \\ ((0.0228463(x-3.15797)-0.00781198)(x-2.4939)+0.00736203)(x-2.93661)+0.625335 & x<2.93661 \\ ((0.00136983(x-3.37932)-0.0065991)(x-2.93661)+0.00217437)(x-3.15797)+0.625816 & x<3.15797 \\ ((0.0159282(x-3.60068)+0.00397825)(x-3.15797)-0.000747116)(x-3.37932)+0.625651 & x<3.37932 \\ ((0.0159282(x-3.15797)+0.00397825)(x-3.37932)+0.0010141)(x-3.60068)+0.625875 & x<3.60068 \\ ((-0.000600503(x-4.92881)-0.00568147)(x-3.60068)-0.00529584)(x-4.70745)+0.620014 & x<4.70745 \\ 0.620014 & \text{True} \end{cases}$$

Section XI - Results comparison of the networks using different activation functions

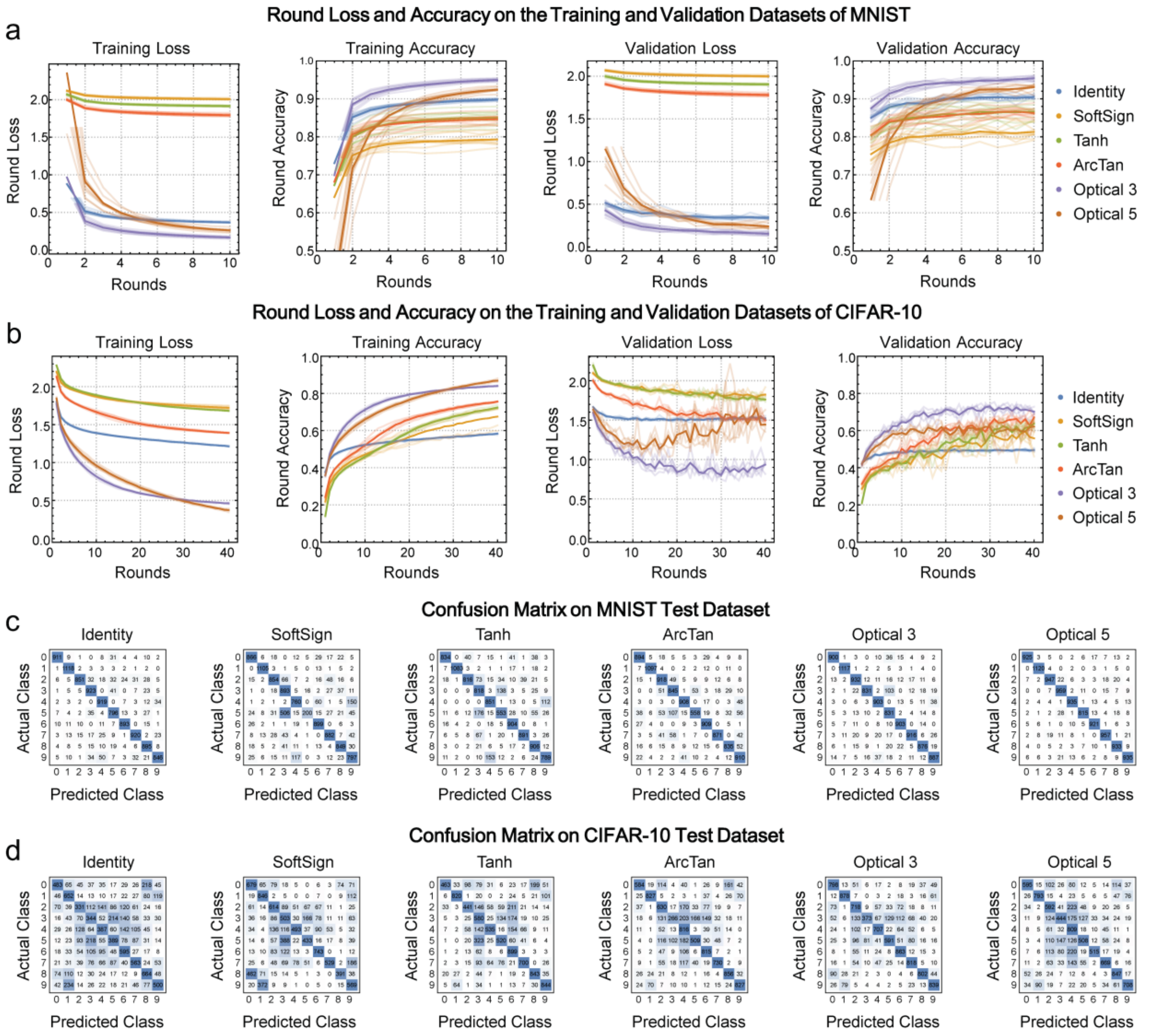

Fig. S15. Performance comparison using different activation functions on MNIST and CIFAR-10 datasets. The round loss and accuracy curves of the complex-valued neural network using three classical activation functions and two optical activation functions with the identity function as a reference on the (a) MNIST and (b) CIFAR-10 datasets. The confusion matrices of the complex-valued neural networks on (c) MNIST and (d) CIFAR-10 datasets, respectively. The confusion matrix is measured on the model with the greatest accuracy of the 5 initializations for each activation function.

The network performances were compared between six different activation functions (1 identity function as a reference and 5 nonlinear functions for comparison) and the results are shown in Fig. S15. The training loss, training accuracy, validation loss and validation accuracy are demonstrated in Fig. S15a and Fig. S15b. The loss curves demonstrate the convergence speed of the network training and the accuracy curves show the training effect. For each activation function, the model is randomly initialized and trained on the MNIST or CIFAR-10 datasets for 5 times (lightly colored lines). The dark solid line is the average result of 5 training sessions. The round loss and accuracy

curves of the complex-valued neural networks use three classical activation functions (SoftSign, Tanh and ArcTan) and two of the optical activation functions (optical 3 and optical 5) with the Identity function as a reference in the training data set and the verification data set with the increasing rounds. For each activation function, the model is randomly initialized and trained on the MNIST dataset for 5 times. For each activation function, the model is randomly initialized and trained on the CIFAR-10 dataset for 5 times. The solid lines in Fig. S15a and Fig. S15b are the average results of 5 training sessions which are plotted in lightly-colored lines. The results using optical activation functions 3 and 5 show much better loss and higher accuracy than the best classical activation function (ArcTan) in both the MNIST and CIFAR-10 datasets. These advantages are due to the transmission falling to zero for larger input values for classical functions. A near-zero transmission will result in zero gradient values, which will drawback the updating of network weights. The confusion matrices in Fig. S15c and Fig. S15d for MNIST and CIFAR-10 datasets are measured on the model with the greatest accuracy of the 5 initializations for each activation function. The confusion matrices are results for 10,000 test data set images, and the optical activation functions still demonstrate better performance than classical activation functions.

Section XII - Training results using additional optical activation functions without/with phase shift

The computation with phase shift is expensive to perform, due to the multiple sine and cosine functions in the process, making backward propagation much slower than that of considering only the transmission rate. The advantage in the performance of our optical activation function might not be the result of the additional phase information in activation, but the result of the activation functions of intensity affected by the phase information. To demonstrate the necessity of the phase information in activation, we removed all phase information in the simulation of the optical activation functions. We trained and tested 5 optical activation functions (Fig. 4 in the main text) without phase on the MNIST and CIFAR-10 datasets using the same methods as mentioned in the main text.

For MNIST dataset, the loss and accuracy curves in the training and validation are demonstrated in Fig. S16. And the confusion matrices are demonstrated in Fig. S17. For CIFAR-10 dataset, loss and accuracy curves in the training and validation are demonstrated in Fig. S18. And the confusion matrices are demonstrated in Fig. S19.

The difference in training results between the five optical activation functions without the phase shift is minimal, although their transmission rate differs significantly.

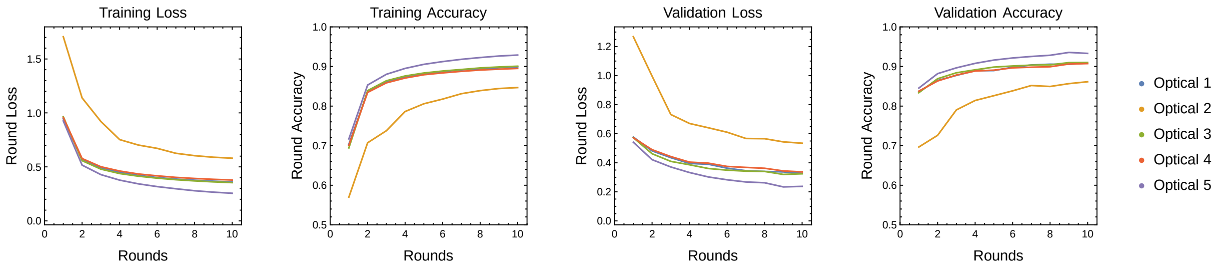

Fig. S16. Round loss and accuracy results using five optical activation functions demonstrated in the main text without phase change, on MNIST dataset.

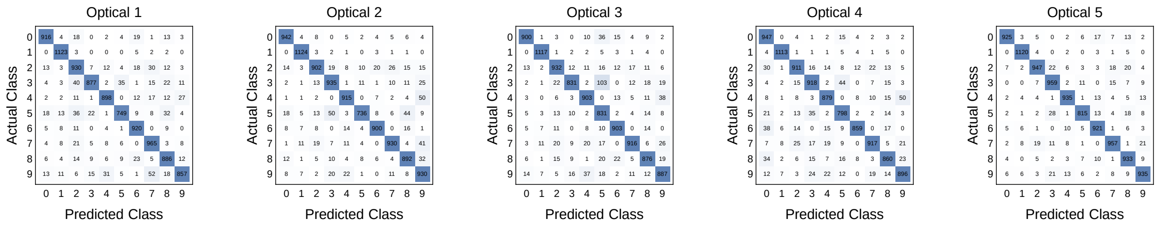

Fig. S17. Confusion matrices for the ONN using five optical activation functions mentioned in the main text without phase information, on test set of MNIST dataset.

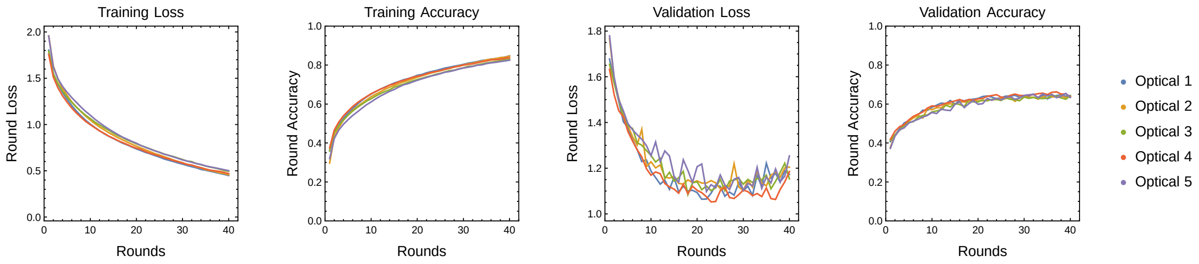

Fig. S18. Training results for five optical activation functions without phase. The figure shows the round loss and accuracy during the training process of the network on CIFAR-10 dataset.

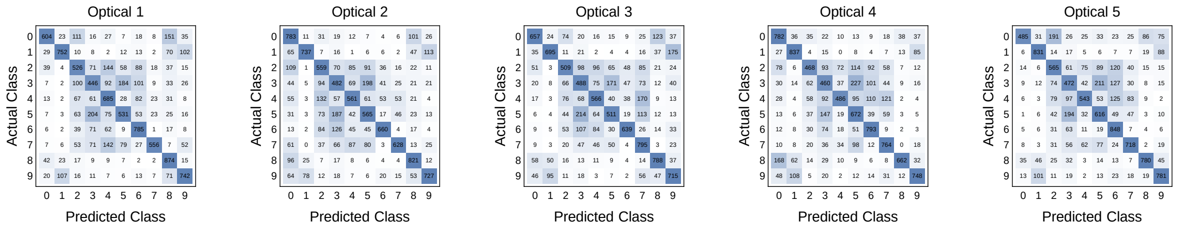

Fig. S19. Testing results for five optical activation functions without phase. The figure shows the confusion matrix of the network on CIFAR-10 dataset test set.

The phase information with respect to the activation is proved to have a significant impact on the training process and the final performance, as shown in Fig. S20, Fig. S21, Fig. S22 and Fig. S23, compared to the five activation functions without the phase shift applied, shown in Fig. S16 – Fig. S19.

Fig. S20 and Fig. S25 show the round loss and accuracy during the network training process on MNIST and CIFAR-10 datasets, respectively. Fig. S21 and Fig. S23. show the confusion matrices of the network on MNIST and CIFAR-10 test sets, respectively.

With the phase shift applied, their performances showed dramatic changes, proving that the phase shift is vital for the activation process.

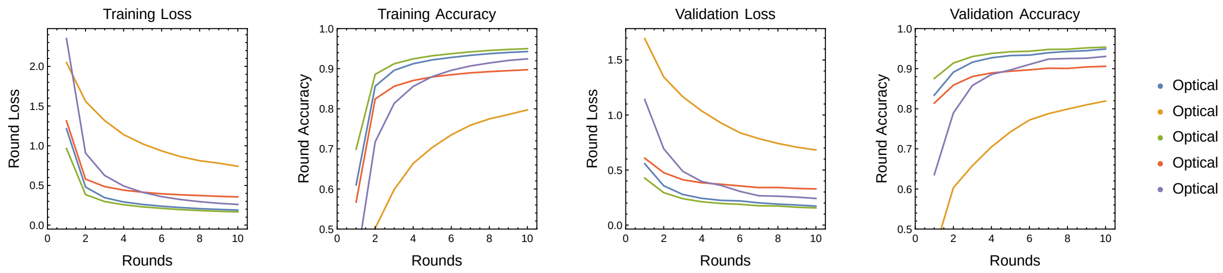

Fig. S20. Training results for five optical activation functions with phase on MNIST dataset.

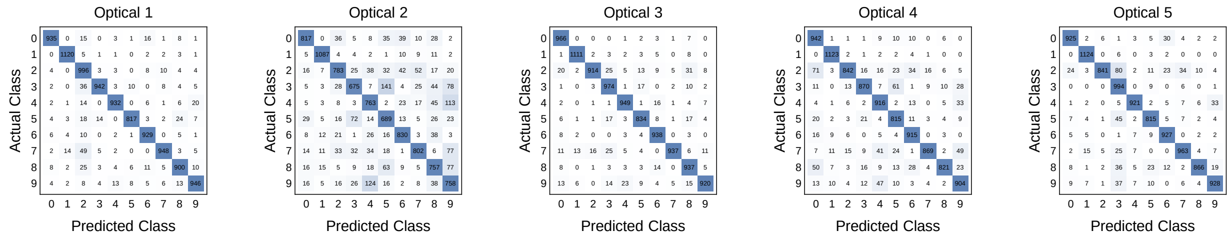

Fig. S21. Testing results for five optical activation functions with phase on MNIST dataset.

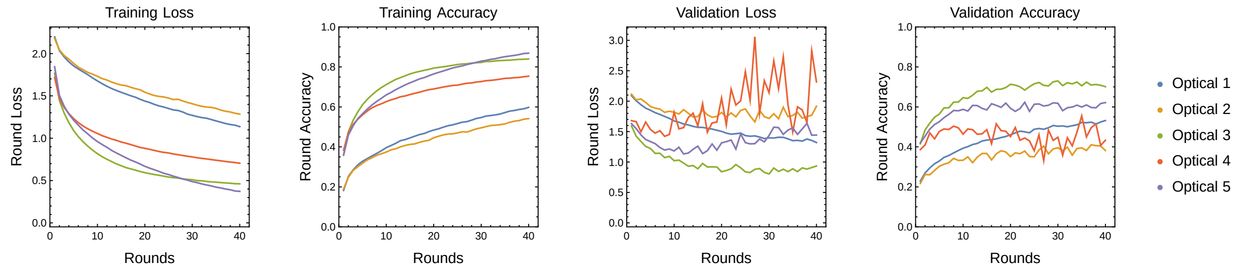

Fig. S22. Training results for five optical activation functions with phase on CIFAR-10 dataset.

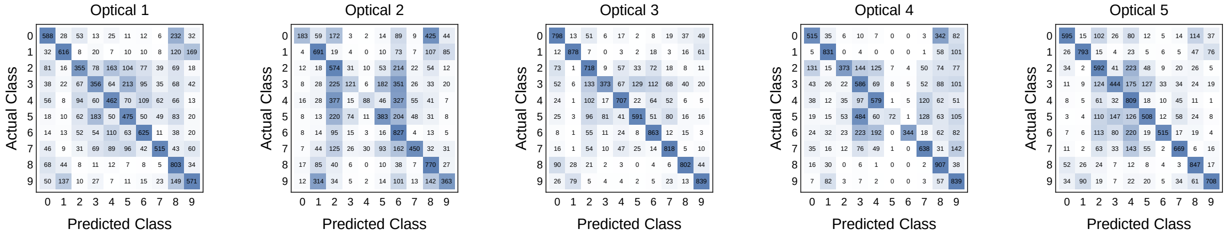

Fig. S23. Testing results for five optical activation functions with phase. The figure shows the confusion matrices of the network on CIFAR-10 dataset test set.

Section XIII - Comparison of the activation maps using different activation functions and optical functions

The activation maps of MNIST and CIFAR-10 datasets using three classical activation functions, including Tanh, ArcTan and Softsign and optical activation function 3 are demonstrated in Fig. S24 and Fig. S25, respectively. Compared with the activation maps of classical activation functions, the optical activation maps display a sparsity of features at a much earlier stage, with other similar differences of the MNIST activation map included.

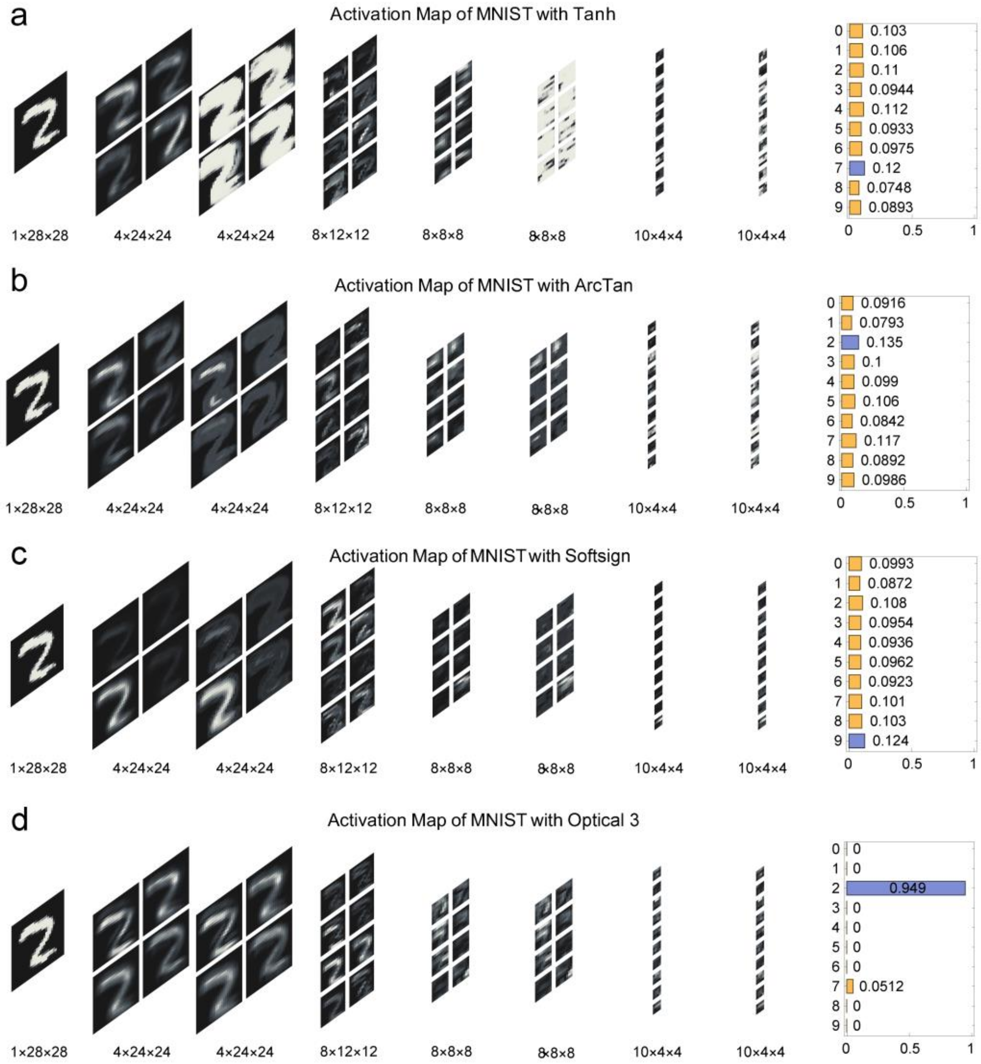

Fig. S24. Visualized activation map trained on MNIST dataset using (a) Tanh, (b) ArcTan, (c) Softsign and (d) Optical 3 activation functions.

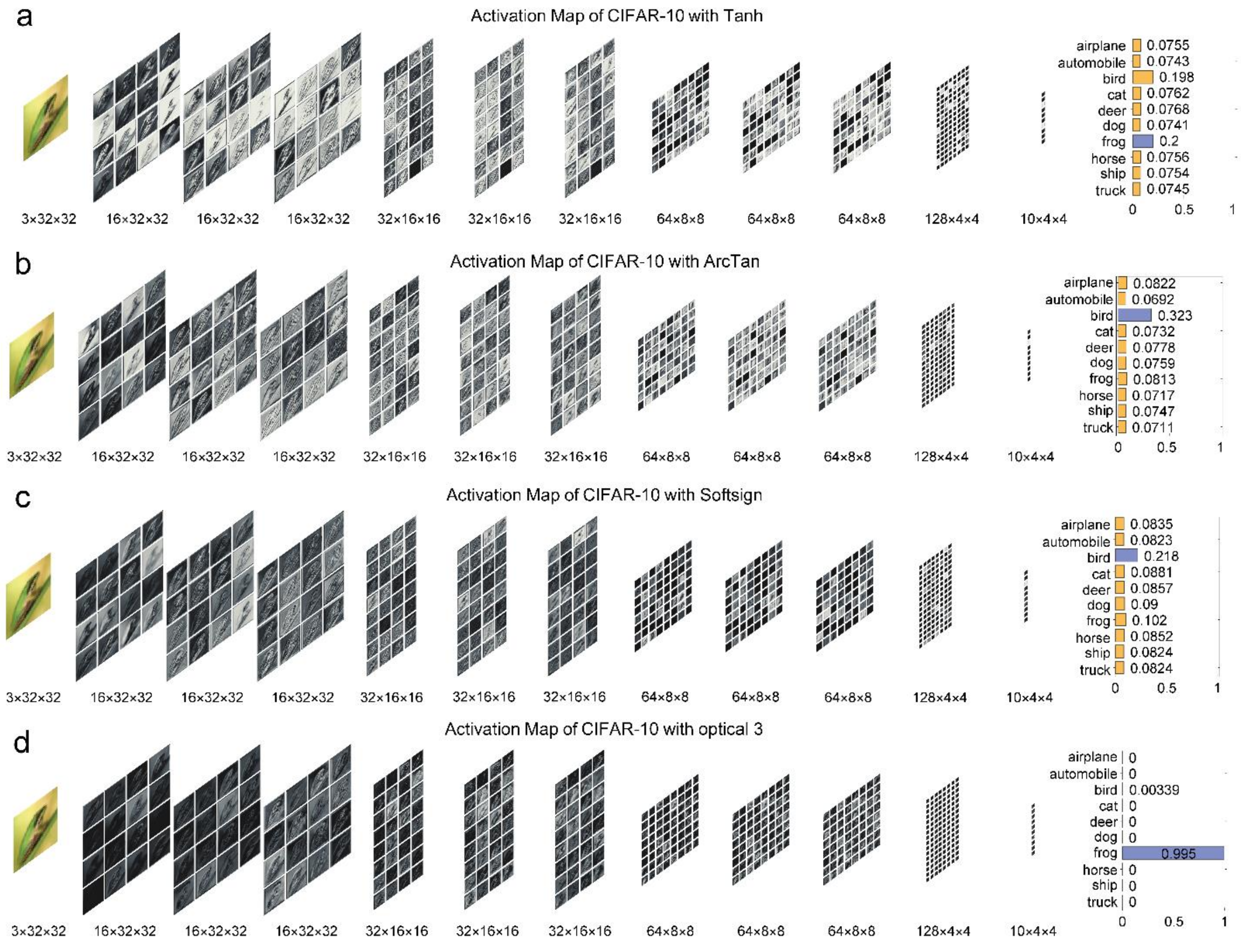

Fig. S25. Visualized activation map trained on CIFAR-10 dataset using (a) Tanh, (b) ArcTan, (c) Softsign and (d) Optical 3 activation functions.

References

[S1]. Ferrari, A. C. & Basko, D. M. Raman spectroscopy as a versatile tool for studying the properties of graphene. *Nat Nanotechnol* **8**, 235-246 (2013)

[S2]. Das, A., et al. Monitoring dopants by Raman scattering in an electrochemically top-gated graphene transistor. *Nat Nanotechnol* **3**, 210-215 (2008)

[S3]. Lee, J. E., Ahn, G., Shim, J., Lee, Y. S. & Ryu, S. Optical separation of mechanical strain from charge doping in graphene. *Nat Commun* **3**, 1024 (2012)

[S4]. Ullah, S., et al. Graphene transfer methods: A review. *Nano Res* **14**, 3756–3772 (2021)

[S5]. Shi, J., et al. 16-channel photonic–electric co-designed silicon transmitter with ultra-low power consumption. *Photon Res* **11**, (2023)

[S6]. Sobu, Y., Simoyama, T., Tanaka, S., Tanaka, Y. & Morito, K. 70 Gbaud Operation of All-Silicon Mach–Zehnder Modulator based on Forward-Biased PIN Diodes and Passive Equalizer. 2019 24th OptoElectronics and Communications Conference (OECC) and 2019 International Conference on Photonics in Switching and Computing (PSC); 2019 7-11 July 2019; 2019. p. 1-3.

[S7]. Zhang, W., et al. Harnessing plasma absorption in silicon MOS ring modulators. *Nat Photonics* **17**, 273-279 (2023)

[S8]. Wang, X., et al. High-speed silicon photonic Mach–Zehnder modulator at 2 μm. *Photonics Res* **9**, 535-540 (2021)

[S9]. Sun, C., et al. High-performance silicon PIN diode switches in the 2-microm wave band. *Opt Lett* **47**, 2758-2761 (2022)

[S10]. Liu, M., et al. A graphene-based broadband optical modulator. *Nature* **474**, 64-67 (2011)

[S11]. Phare, C. T., Daniel Lee, Y.-H., Cardenas, J. & Lipson, M. Graphene electro-optic modulator with 30 GHz bandwidth. *Nat Photonics* **9**, 511-514 (2015)

[S12]. Sorianello, V., et al. Graphene–silicon phase modulators with gigahertz bandwidth. *Nat Photonics* **12**, 40-44 (2017)

[S13]. Wang, Y., et al. Ultrahigh-speed graphene-based optical coherent receiver. *Nat Commun* **12**, 5076 (2021)

[S14]. Gan, X., et al. Chip-integrated ultrafast graphene photodetector with high responsivity. *Nat Photonics* **7**, 883-887 (2013)

[S15]. Wang, X., Cheng, Z., Xu, K., Tsang, H. K. & Xu, J.-B. High-responsivity graphene/silicon-heterostructure waveguide photodetectors. *Nat Photonics* **7**, 888-891 (2013)

[S16]. Pospischil, A., et al. CMOS-compatible graphene photodetector covering all optical communication bands. *Nature Photonics* **7**, 892-896 (2013)

[S17]. Schuler, S., et al. High-responsivity graphene photodetectors integrated on silicon microring resonators. *Nat Commun* **12**, 3733 (2021)

[S18]. Guo, J., et al. High-performance silicon-graphene hybrid plasmonic waveguide photodetectors beyond 1.55 mum. *Light Sci Appl* **9**, 29 (2020)

[S19]. Hagan, D. E., Ye, M., Wang, P., Cartledge, J. C. & Knights, A. P. High-speed performance of a TDFA-band micro-ring resonator modulator and detector. *Opt Express* **28**, 16845-16856 (2020)

[S20]. Youngblood, N., Anugrah, Y., Ma, R., Koester, S. J. & Li, M. Multifunctional graphene optical modulator and photodetector integrated on silicon waveguides. *Nano Lett* **14**, 2741-2746 (2014)

[S21]. Lin, X., et al. All-optical machine learning using diffractive deep neural networks. *Science* **361**, 1004-+ (2018)

[S22]. Zhou, T., et al. Large-scale neuromorphic optoelectronic computing with a reconfigurable diffractive processing unit. *Nat Photonics* **15**, 367-373 (2021)

[S23]. Zeng, H., Fan, J., Zhang, Y., Su, Y., Qiu, C. & Gao, W. Graphene plasmonic spatial light modulator for reconfigurable diffractive optical neural networks. *Opt Express* **30**, 12712-12721 (2022)

[S24]. Martinez-Martinez, R., Islam, M. M., Krishnaprasad, A. & Roy, T. Graphene-oxide interface for optoelectronic synapse application. *Sci Rep* **12**, 5880 (2022)

[S25]. Feldmann, J., Youngblood, N., Wright, C. D., Bhaskaran, H. & Pernice, W. H. P. All-optical spiking neurosynaptic networks with self-learning capabilities. *Nature* **569**, 208-214 (2019)
[S26]. Yu, T., et al. All-Chalcogenide Programmable All-Optical Deep Neural Networks. Preprint at https://ui.adsabs.harvard.edu/abs/2021arXiv210210398Y (2021).
[S27]. Liao, K., et al. Matrix eigenvalue solver based on reconfigurable photonic neural network. *Nanophotonics* **11**, 4089-4099 (2022)
[S28]. Shi, Y., et al. Nonlinear germanium-silicon photodiode for activation and monitoring in photonic neuromorphic networks. *Nat Commun* **13**, (2022)
[S29]. Amin, R., et al. ITO-based electro-absorption modulator for photonic neural activation function. *APL Mater* **7**, 081112 (2019)
[S30]. Amin, R., et al. An ITO–graphene heterojunction integrated absorption modulator on Si-photonics for neuromorphic nonlinear activation. *APL Photonics* **6**, 120801 (2021)
[S31]. Jha, A., Huang, C. & Prucnal, P. R. Reconfigurable all-optical nonlinear activation functions for neuromorphic photonics. *Opt Lett* **45**, 4819-4822 (2020)
[S32]. Pour Fard, M. M., et al. Experimental realization of arbitrary activation functions for optical neural networks. *Opt Express* **28**, 12138-12148 (2020)
[S33]. Huang, Y., Wang, W., Qiao, L., Hu, X. & Chu, T. Programmable low-threshold optical nonlinear activation functions for photonic neural networks. *Opt Lett* **47**, 1810-1813 (2022)
[S34]. Ashtiani, F., Geers, A. J. & Aflatouni, F. An on-chip photonic deep neural network for image classification. *Nature* **606**, 501-506 (2022)
[S35]. Farshid Ashtiani, Alexander J. Geers & Aflatouni, F. Single-chip photonic deep neural network for instantaneous image classification. Preprint at https://arxiv.org/abs/2106.11747 (2022).
[S36]. Xu, Z., et al. Reconfigurable nonlinear photonic activation function for photonic neural network based on non-volatile opto-resistive RAM switch. *Light Sci Appl* **11**, 288 (2022)
[S37]. Williamson, I. A. D., Hughes, T. W., Minkov, M., Bartlett, B., Pai, S. & Fan, S. Reprogrammable Electro-Optic Nonlinear Activation Functions for Optical Neural Networks. *IEEE J Sel Top Quantum Electron* **26**, 1-12 (2020)
[S38]. Schwelb, O. Transmission, Group Delay, and Dispersion in Single-Ring Optical Resonators and Add/Drop Filters—A Tutorial Overview. *Journal of Lightwave Technology* **22**, 1380-1394 (2004)
[S39]. Yariv, A. Universal relations for coupling of optical power between microresonators and dielectric waveguides. *Electronics Letters* **36**, 321-322 (2000)
[S40]. Hirose, A. *Complex-Valued Neural Networks*, 2013.
[S41]. Trabelsi, C., et al. Deep complex networks. Preprint at http://arxiv.org/abs/1705.09792 (2018).
[S42]. Kingma, D. P. & Ba, J. Adam: A Method for Stochastic Optimization. Preprint at http://arxiv.org/abs/1412.6980 (2017).
[S43]. He, K., Zhang, X., Ren, S. & Sun, J. Delving Deep into Rectifiers: Surpassing Human-Level Performance on ImageNet Classification. Preprint at http://arxiv.org/abs/1502.01852 (2015).
[S44]. Xu, B., Wang, N., Chen, T. & Li, M. Empirical Evaluation of Rectified Activations in Convolutional Network. Preprint at http://arxiv.org/abs/1505.00853 (2015).
[S45]. Clevert, D.-A., Unterthiner, T. & Hochreiter, S. Fast and accurate deep network learning by exponential linear units (ELUs). Preprint at http://arxiv.org/abs/1511.07289 (2016).
[S46]. Courbariaux, M., Bengio, Y. & David, J.-P. BinaryConnect: Training Deep Neural Networks with binary weights during propagations. Preprint at http://arxiv.org/abs/1511.00363 (2016).
